# A sporadic Parkinson’s disease model via silencing of the ubiquitin–proteasome/E3 ligase component, SKP1A

**DOI:** 10.1007/s00702-023-02687-6

**Published:** 2023-08-29

**Authors:** Tali Fishman-Jacob, Moussa B. H. Youdim

**Affiliations:** Youdim Pharmaceutical Ltd, New Northern Industrial Park, 1 Ha- Tsmikha St, Stern Building, Fl-3, P. O. Box 72, 2069207 Yokneam, Israel

**Keywords:** Novel Parkinson's Disease model, SKP1A knock down, Dopaminergic degeneration, SKP1A manipulation as therapeutic approach, Cullin, F-box protien, ubiquitin proteasome ligases

## Abstract

Our and other’s laboratory microarray-derived transcriptomic studies in human PD substantia nigra pars compacta (SNpc) samples have opened an avenue to concentrate on potential gene intersections or cross-talks along the dopaminergic (DAergic) neurodegenerative cascade in sporadic PD (SPD). One emerging gene candidate identified was *SKP1A* (p19, S-phase kinase-associated protein 1A), found significantly decreased in the SNpc as confirmed later at the protein level. *SKP1* is part of the Skp1, Cullin 1, F-box protein (SCF) complex, the largest known class of sophisticated ubiquitin–proteasome/E3-ligases and was found to directly interact with *FBXO7*, a gene defective in *PARK15*-linked PD. This finding has led us to the hypothesis that a targeted site-specific reduction of Skp1 levels in DAergic neuronal cell culture and animal systems may result in a progressive loss of DAergic neurons and hopefully recreate motor disabilities in animals. The second premise considers the possibility that both intrinsic and extrinsic factors (e.g., manipulation of selected genes and mitochondria impairing toxins), alleged to play central roles in DAergic neurodegeneration in PD, may act in concert as modifiers of Skp1 deficiency-induced phenotype alterations (‘dual-hit’ hypothesis of neurodegeneration). To examine a possible role of Skp1 in DAergic phenotype, we have initially knocked down the expression of *SKP1A* gene in an embryonic mouse SN-derived cell line (*SN4741*) with short hairpin RNA (shRNA) lentiviruses (LVs). The deficiency of *SKP1A* closely recapitulated cardinal features of the DAergic pathology of human PD, such as decreased expression of DAergic phenotypic markers and cell cycle aberrations. Furthermore, the knocked down cells displayed a lethal phenotype when induced to differentiate exhibiting proteinaceous round inclusion structures, which were almost identical in composition to human Lewy bodies, a hallmark of PD. These findings support a role for Skp1 in neuronal phenotype, survival, and differentiation. The identification of Skp1 as a key player in DAergic neuron function suggested that a targeted site-specific reduction of Skp1 levels in mice SNpc may result in a progressive loss of DAergic neurons and terminal projections in the striatum. The injected LV *SKP1*shRNA to mouse SN resulted in decreased expression of Skp1 protein levels within DAergic neurons and loss of tyrosine hydroxylase immunoreactivity (TH-IR) in both SNpc and striatum that was accompanied by time-dependent motor disabilities. The reduction of the vertical movements, that is rearing, may be reminiscent of the early occurrence of hypokinesia and axial, postural instability in PD. According to the ‘dual-hit’ hypothesis of neurodegenerative diseases, it is predicted that gene–gene and/or gene–environmental factors would act in concert or sequentially to propagate the pathological process of PD. Our findings are compatible with this conjecture showing that the genetic vulnerability caused by knock down of *SKP1A* renders DAergic *SN4741* cells especially sensitive to genetic reduction of Aldh1 and exposure to the external stressors MPP^+^ and DA, which have been implicated in PD pathology. Future consideration should be given in manipulation *SKP1A* expression as therapeutic window, via its induction genetically or pharmacological, to prevent degeneration of the nigra striatal dopamine neurons, since UPS is defective.

## Introduction

### Parkinson’s disease

Parkinson’s disease (PD) may be the second most common neurodegenerative disease after Alzheimer’s disease (AD), featuring an incidence that increases with age and a higher prevalence in the male population. PD is a multifactorial disorder, and several factors related to genes, age, sex, and environment may increase the risk to contract the disease. Indeed, it is assumed that PD and other neurodegenerative disorders are caused by a complex interaction between genetic predisposition and endotoxins or neurotoxins as stated by the multifactorial or “dual-hit” hypothesis (Boger et al. 2010). A primary pathogenic event is associated with degeneration of the nigro-striatal dopamine (DA) producing neurons associated with the presence of intracytoplasmatic inclusions of ubiquitin and α-synuclein denominated Lewy bodies (LB), a pathological hallmark of PD (Dauer and Przedborski 2003). PD is characterized by severe clinical motor symptoms, including uncontrollable tremor, rigidity, postural instability, and slowness or absence of voluntary movement. The most common treatment is levodopa (l-DOPA), which makes up for the loss of DA, though no disease-modifying treatment exists that could halt or delay the disease progression. It is interesting to note that the motor symptoms characterizing the disease are manifested once degeneration of the dopaminergic (DAergic) nigro-striatal pathway has reached at least 70–80%. The current concept, regarding PD and other neurodegenerative disorders, considers them diseases of multiple etiological nature where several mechanisms are implicated in a cascade/s of events involving many biochemical and signaling pathways (Mandel et al. 2003; Nagatsu et al. 2023; Riederer et al. 2023; Conway et al. 2022). These include the impairment in mitochondrial activity, a failure of the ubiquitin–proteasome system (UPS) to adequately remove abnormal proteins, and a general environment of oxidative stress (OS) (Langston et al. 1984; Heikkila et al. 1984; Rosner et al. 2008), that might cause a derangement in cell cycle control. The presence of oxygen reactive species (ROS) would exacerbate protein misfolding and the demand for their disposition by the UPS, the activity of which is, however, impaired in PD. This would result in the propagation of a vicious cycle which may be self-sustaining.

It is also now appreciated that PD is associated with extensive non-DAergic pathology, which involves several other neurotransmitters, including acetylcholine, noradrenaline, glutamate, and adenosine (Schapira et al. 2006). These abnormalities are responsible for the non-motor symptoms of PD that may even precede the onset of motor symptoms, sometimes by years and are often considered more debilitating to the afflicted patients. Common manifestations are freezing, falling, cognitive decline, anosmia (loss of sense of smell), problems with gastrointestinal motility, sleep disturbances, sympathetic denervation, anxiety, and depression (Langston 2006).

### Sporadic vs. familial PD

**Fig. 1:**
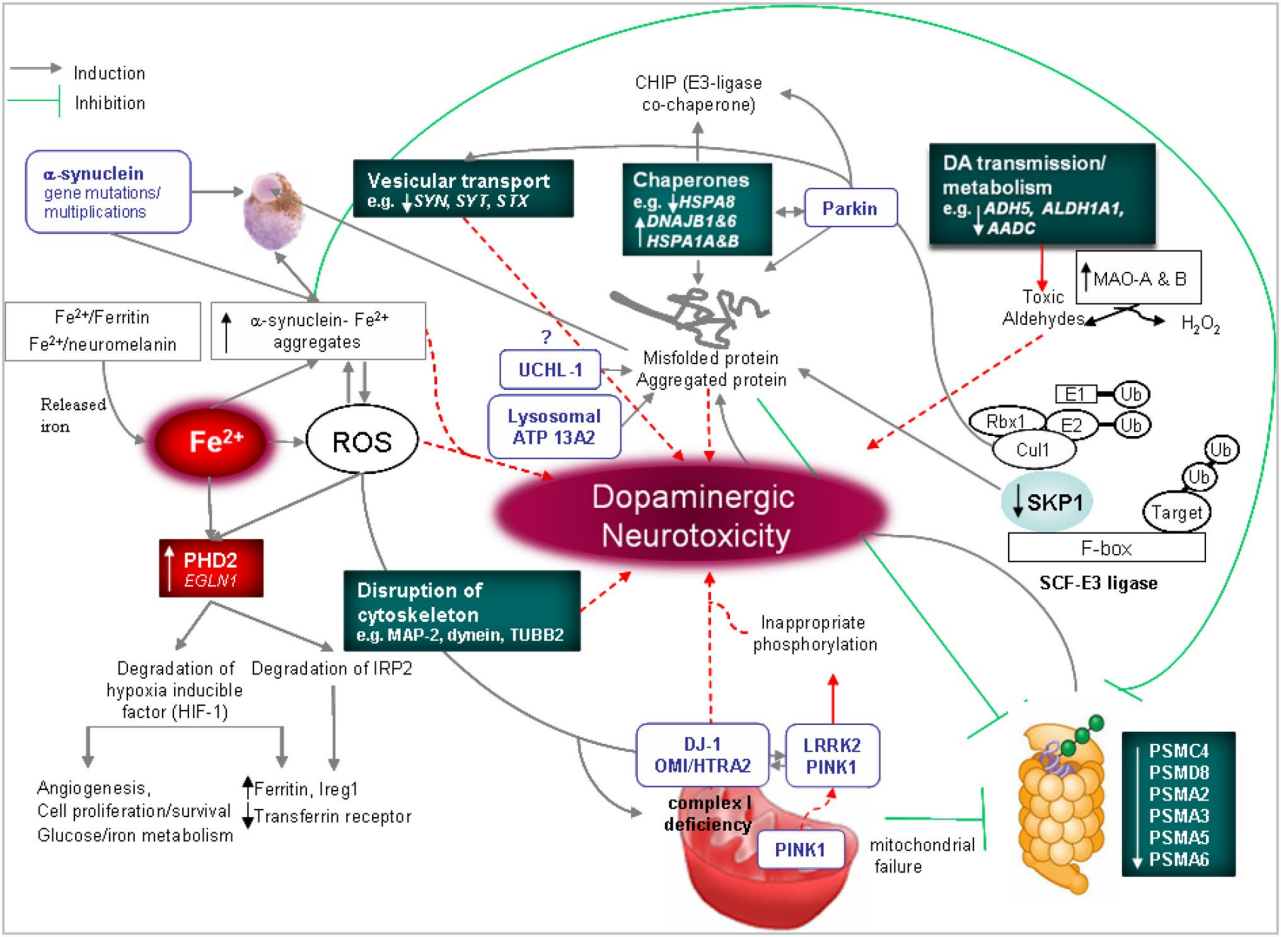
Schematic diagram of major gene and neurochemical alterations in human SNpc of PD (Fishman-Jacob et al. 2010)

The identification of mutations linked to heritable forms of PD and the implementation of microarray-based gene expression profiling during the past decade has provided additional clues on how the disease initiates and progresses as well as potential molecular targets that may be of relevance to both familial and sporadic PD (Fig. 1) (Grunblatt et al. 2004; Hauser et al. 2005; Miller et al. 2006; Moran et al. 2006; Zhang et al. 2005; Gasser 2009; Cherian et al. 2023; Nott and Holtman 2023). There is a recognized consensus that in both the genetic and sporadic cases of PD, there is a crucial implication of mitochondria and UPS dysfunction that expresses itself with excess production of ROS, protein misfolding, and aggregation into inclusion bodies. However, the precise identity of the pivotal genes involved in the neurotoxic cascade pathways leading to the death of the DAergic neurons in sporadic PD (SPD) is still unknown (Mandel et al. 2009; Cherian et al. 2023). In the past decade, model-based linkage analysis in larger pedigrees has identified genetic mutations in LBs PD and many forms of atypical Parkinsonism; pathogenic mutations have been confirmed to date in at least 11 genes and several more remain to be identified (see Table 1, (Farrer 2006)). The first gene to be linked to the rare form of the disease was *PARK1* (Polymeropoulos et al. 1997; Patel et al. 2022; Connelly et al. 2023; Pereira et al. 2023). The mutation identified in the α-synuclein gene was a single point mutation (A53T), and subsequently, several other autosomal dominantly inherited Parkinsonism was also identified. The rare autosomal-dominant Parkinsonian syndrome is clinically as well as pathologically similar to SPD (Golbe et al. 1990; Kruger et al. 1998; Polymeropoulos et al. 1997). α-Synuclein is considered a natively unfolded protein with a high propensity to aggregate, with the end product of abnormal filamentous inclusions (Cookson 2004). Independently to α-synuclein and 1 year later, gene mutations in parkin (Kitada et al. 1998) and ubiquitin c-terminal hydrolase-L1 (*UCHL-1*) (Leroy et al. 1998), which are capable of impairing the activity of the UPS, have been described in rare forms of hereditary PD. UCHL-1 has ubiquitin hydrolase and ligase activities removing ubiquitin from processed amino-acid fragments and as such is responsible for impairment of protein degradation. Loss-of-function mutations in the gene-encoding parkin cause recessively inherited Parkinsonism (Kitada et al. 1998). The gene product parkin (*PARK2*) is an E3 ligase involved in targeting certain substrate proteins for degradation by the proteasome. Most patients with this form of the disease lack of LBs, suggesting that parkin is required for their formation/maintenance (Shimura et al. 2000). Two additional loss-of-function mutations, DJ-1 (Bonifati et al. 2003) and PTEN-induced kinase 1 (PINK-1) (Valente et al. 2004), result in an early age of onset (typically, at less than 40 years of age) and a disease that is l-DOPA responsive with slow progression (Mata et al. 2004; Abou-Sleiman et al. 2004). They have been proposed to play a role in cellular response to OS and in mitochondrial function. More recently, another mutation was found in autosomal-dominant Parkinsonism families in a gene encoding a large, multifunctional protein, Leucine-rich repeat kinase 2 (LRRK2) (Zimprich et al. 2004; Paisan-Ruiz et al. 2004). The PARK8 locus was originally mapped as an autosomal-dominant trait in a Japanese family with asymmetrical, l-DOPA-responsive, late-onset PD (Funayama et al. 2002). The discovery was subsequently confirmed by linkage in several families and sequencing of the gene has now revealed many pathogenic amino-acid substitutions (Kachergus et al. 2005; Paisan-Ruiz et al. 2005; Funayama et al. 2005; Mata et al. 2005). The LRRK2 6099G > A (Gly2019Ser) mutation leads to the most frequent substitution in Caucasians, which typically explains 0.5–2.0% of cases of SPD and 5% of familial Parkinsonism (Kachergus et al. 2005; Gilks et al. 2005; Di Fonzo et al. 2005). However, in Ashkenazi Jews and North African Arab populations, this mutation might account for 18–30% of PD cases (Ozelius et al. 2006; Lesage et al. 2006). The clinical features resemble the idiopathic variant with a tremor, slow progression of symptoms, and no observation of cognitive disturbance (Gosal et al. 2005). In addition, mutations have been identified in a number of genes for which the pattern of inheritance is not clear or for which there has been no independent confirmation. These include the *NR4A2*, synphylin-1, and Htra serine peptidase 2 (Htra2) (Le et al. 2003; Leroy et al. 1998; Marx et al. 2003; Strauss et al. 2005). Htra2, also known as Omi, is a ubiquitously expressed protein, which is localized to the mitochondrial intermembrane space but can be released into the cytosol after apoptotic stimuli (Hegde et al. 2002; Martins et al. 2002). Mouse model entirely lacking expression of *HTRA2*/PARK13/Omi shows no evidence of reduced rates of cell death but on the contrary suffers loss of a population of neurons in the striatum, resulting in a neurodegenerative disorder with a parkinsonian phenotype that leads to death of the mice around 30 days after birth (Martins et al. 2004). In addition to these, a gene causing Kufor–Rakeb syndrome was identified in the *PARK9* locus (Ramirez et al. 2006). Although initially thought to be a PD locus, mutations in *ATP13A2* are now considered to produce the parkinsonian features associated with Kufor–Rakeb syndrome following striatal degeneration. The syndrome typically manifests with behavioral problems, akinetic-rigidity, pyramidal tract dysfunction, supranuclear gaze paresis, and dementia (Vilarino-Guell et al. 2009). *PLA2G6* was also reported recently as the causative gene for *PARK14*, a form of autosomal recessive early onset dystonia–parkinsonism. The two reported unrelated families with homozygous *PLA2G6* mutation had early onset L-dopa-responsive dystonia–parkinsonism, pyramidal signs, and cognitive/psychiatric features, together with mild generalized cerebral atrophy on MRI but no iron accumulation (Paisan-Ruiz et al. 2009). Furthermore, three novel pathogenic *FBXO7*/*PARK15* mutations in two families were recently reported, showing unambiguously that recessive mutations in the gene encoding the F-box protein 7,*FBXO7 *(Randle and Laman 2017; Joseph et al. 2019; Zhong et al. 2022; Deng et al. 2013) cause a neurodegenerative disease with early onset, parkinsonian–pyramidal phenotype (Di Fonzo et al. 2009). Additional loci have been identified as segregating in families with PD, but the genes have yet to be identified (Toulouse and Sullivan 2008). Although none of the genes implicated in the genetic forms of PD has been shown differentially expressed in the expression profiling studies of human PD substantia nigra (SN) (Grunblatt et al. 2004; Hauser et al. 2005; Miller et al. 2006; Moran et al. 2006; Zhang et al. 2005; Gasser 2009), the putative role/biological process accredited to the majority of the PD-linked genes support the belie that both familial and sporadic forms of PD converge primarily in cascade of impairments in protein handling/degradation mechanisms previously known to be involved in the pathophysiology of the disease, OS damage, mitochondrial dysfunction, auto-oxidation or monoamine oxidase (MAO) oxidation of DA, and excessive iron accumulation in the substantia nigra pars compacta (SNpc). Notably, the vast majority of the genes identified by all the transcriptomic studies include novel gene products in neurodegeneration. It seems plausible that the PD causing genes either converge into a common pathway or participate in parallel signaling cascades that are shared by both the familial and sporadic forms. The above studies have opened an avenue to concentrate on potential gene intersections or cross-talks along the DAergic neurodegenerative cascade in both forms of the disease.

**Table 1:** Identified genes linked to familial PD

Locus	Gene/protein	Chromosome location	Inheritance	Suggested function
*PARK 1/4*	SNCA/ α-synuclein	4q21-q23	AD	Synaptic function/vesicle trafficking
*PARK 2*	PARK2/ Parkin	6q25-q27	AR	E3 ubiquitin ligase
*PARK 5*	UCHL1	4p14	AD	Ubiquitin C-terminal
*PARK 6*	PINKI	1p35-p36	AR	Mitochondrial serine/threonine kinase
*PARK 7*	PARK7/ DJ-1	1p36	AR	Chaperone, oxidative stress response
*PARK 8*	LRRK2/dardarin	12p11.2-q13.1	AD	Protein kinase
*PARK 9*	ATP13 A2	1p36	AR	Lysosomal type 5 ATPase
*PARK 13*	OMI/HTRA2	2p12	AD	Serine protease
*PARK14*	PLA2G6	22q13.1	AR	A2 phospholipase
*PARK 15*	FBXO7	22q12-q13	AR	SCF E3 ubiquitin ligase component
Not assigned	NR4A2/NURR1	2q22-q23	Unknown	Probable nuclear receptor
Not assigned	Synphilin-1	5q23.1-q23.3	Unknown	Synaptic function, protein degradation

*AD autosomal dominant, *AR* autosomal recessive

### Genome-wide association studies (GWAS)

In addition to the familial PD-linked genes, several genomic loci have been designated in linkage studies as either being linked or influencing the age of onset of PD, such as microtubule-associated protein tau (*MAPT*) and ubiquitin carboxyl-terminal esterase L1 (*UCHL1*) [for review of candidate genes studied today, see (Rosner et al. 2008; Farrer 2006; Wider et al. 2010)]. However, linkage analysis is less well suited for the more common complex disorders such as PD and Alzheimer’s disease (AD). Instead, large-scale genome-wide association studies (GWAS) have become increasingly applied to identify regions of the genome influencing the risk of PD. A recent GWAS in PD revealed genetic variations in *SNCA* and at the *MAPT* locus that confirmed them as major risk loci underlying PD across populations (Simon-Sanchez et al. 2009). The authors also provided evidence supporting the role of common variability around *LRRK2* in modulating risk for PD. More recently, 11 loci that surpassed the threshold for genome-wide significance (*p* < 5 × 10( – 8) were recognized. Six were previously identified loci (*MAPT*, *SNCA*, *HLA-DRB5*, *BST1*, *GAK*, and *LRRK2*) and changes in expression and DNA methylation with risk alleles were newly identified at five additional loci (*ACMSD*, *STK39*, *MCCC1/LAMP3*, *SYT11*, and CCDC62/*HIP1R*) (Nalls et al. 2010). GWAS promise to extend genome coverage to reveal common PD susceptibility genes and shed light on the (likely) myriad genetic factors involved in this complex.

### Genetic and toxin-based models of PD

Within the past 40 years, discoveries of environmental and monogenetic forms of Parkinsonism have shaped the direction of PD research and development of experimental systems to investigate underlying mechanisms of cellular degeneration and explore new therapeutic approaches, aimed at slowing or stopping disease progression. The discovery of neurotoxins that selectively lesion the same area of the brain as seen in PD after cerebral injection or systemic administration [e.g., hydroxydopamine (6-OHDA), N-methyl,1,2,4,6-tetrahydropyridine (MPTP)] have given birth to new means to induce models for PD (Ungerstedt 1968; Langston et al. 1984; Heikkila et al. 1984) and opened up the possibility that similar compounds in the environment might actually play a causative role in the sporadic/idiopathic disease itself. The second major impetus for the development of new models for PD has resulted from the identification of several different monogenetic forms of Parkinsonism (Rosner et al. 2008). This began with discovery of a mutation in the gene-encoding alpha α-synuclein (Polymeropoulos et al. 1997), demonstrating for the first time that a specific mutation can cause a well-defined form of Parkinsonism. Transgenic/knockout mice models derived from the monogenetic forms of Parkinsonism have made contributions to our current understanding of the pathogenesis of PD, highlighting potential mechanistic pathways for future therapeutic intervention.

In spite of the huge progress in pathogenic modeling using a range of toxins and transgenic, knockout, viral-based models of gene defects in familial PD (FPD) and mutant rodents, none of the existing PD animal models recapitulate most key features of the disease, namely derangements in DAergic synaptic transmission, selective neurodegeneration, neurochemical deficits, α-synuclein-positive neuropathology, involvement of extra-nigral areas, the motor and non-motor deficits, and drug responsiveness as seen in humans (Jenner 2008; Manning-Bog and Langston 2007). MPTP, rotenone, and 6-OHDA have been important in understanding mechanisms related to denervation in the basal ganglia, since they may cause more than 80% loss of SN DAergic neurons and terminals. However, the damage is acute (though recently chronic pump administration of low doses has been advocated), not progressive, and not always accompanied by strong behavioral symptoms.

On the contrary, genetic (mutated)-based models have produced generally mild and sometimes inconsistent phenotypes without dramatic lesion of the nigra or behavioral changes. Although the recently reported mutant *LRRK2* mice model of PD shows an age-dependent and l-DOPA-responsive slowness of movement, neither DA neuron loss nor presence of LB-like α-synuclein inclusions was visible in the SN (Li et al. 2009). This may indicate that neurodegeneration in PD may not result from a single gene defect but from combination of aberrant gene products. It is obvious that neither the toxin-based nor the FPD-linked gene models will contribute to the identification of the final cascade of molecular events that result in degeneration of DA neurons in both FPD and SPD (Fig. 1). It sounds reasonable that novel reliable models of idiopathic PD should consider a careful selection of genes identified as significantly affected in both FPD and SPD.

### Impairment of DA neurotransmission in PD

DAergic neurotransmission is mediated by the vesicular release of DA, i.e., DA exocytosis. DA exocytosis and its modulation are generally believed to affect neuronal communication, development, maintenance, and survival, and contribute to extracellular DA levels in the brain (Westerink 2006). Under normal physiological conditions, the DA transporter (DAT) and vesicular monoamine transporter-2 (VMAT2) are key components in the regulation of DA disposition in the synapse and cytosol. Following vesicular DA release into the synapse, the DAT transports DA from the peri-synaptic area into the cytosol of the presynaptic nerve terminal of DA neurons. The VMAT2 transports cytosolic and newly synthesized DA into synaptic vesicles. Sequestration of DA into these vesicles makes the transmitter available for synaptic release in response to appropriate stimulation. It also provides an environment to protect against the intracellular production of ROS that can result from DA oxidation (Riddle et al. 2005).

Loss of cells in the SN of PD patients results in profound DA depletion in the striatum, the lateral nigral projections to putamen, being most affected. The decline of DA function in PD has encouraged the development of surrogate biological markers that can be followed up during the course of the disease. These include positron emission tomography (PET) and single photon emission computed tomography (SPECT), that can measure striatal DA terminal function in vivo as reflected by DA storage capacity and transporter binding (Brooks 2004; Brooks and Piccini 2006). Studies on imaging of the DAergic system suggest a curvilinear course of progression which starts at different time points in different striatal regions and which levels off after several years of disease duration. There is an annual 4–12% loss of DA terminal function in early PD and a preclinical disease window of only a few years (Brooks 1998; Brucke et al. 2000). Therefore, functional imaging in adjunct to clinical assessment could provide valuable information on disease status.

Recent large transcriptomic microarray studies with human post-mortem SN from PD individuals support and extend these findings providing novel molecular players in the neurodegeneration cascade in PD (Grunblatt et al. 2004; Hauser et al. 2005; Miller et al. 2006; Moran et al. 2006; Zhang et al. 2005; Gasser 2009). The reports demonstrated down-regulation of genes expressing DAergic phenotype [i.e., DAT, aromatic amino-acid decarboxylase (AADC), AMP-regulated phosphoprotein (*ARPP-21*), aldehyde dehydrogenase 1 family, member A1 (*ALDH1A1*) and VMAT2)], vesicle trafficking, and synaptic transmission [i.e., synaptotagmin 1 (SYT1), syntaxin-1A (STX1A), synaptogyrin 3 (SYNGR3), and N-ethyl maleimide-sensitive factor (NSF)]. The fact that similar pathway-related genes have been identified by separate microarray examinations, employing different brain samples and experimental conditions, supports the validity of the findings. Furthermore, a number of the identified genes could serve as potential molecular biomarkers for the disease [expert opinion in (Silvia Mandel 2007)].

### UPS dysfunction in PD

Protein degradation is one of the essential mechanisms regulating levels of cellular proteins involved in crucial cellular processes such as cell cycle, development and growth, transcription, cell signaling, and antigen processing (DeMartino and Slaughter 1999). The UPS is responsible for the detoxification and targeting of damaged proteins for degradation (Coux et al. 1996; Rock et al. 1994; Jang and Chung 2022; Licchesi et al 2020; Di Dominico and Lanzillotta 2022), except membrane and extracellular proteins, which after endocytosis are degraded within the lysosomes (Sherman and Goldberg 2001). Proteins to be degraded are first marked by covalent attachment of a polyubiquitin chain to a lysine residue on the substrate and then are degraded by a large proteolytic complex, the 26S proteasome. Polyubiquitination of a target protein is required to ensure specificity and activate the ubiquitin moiety. Parkin implicated in FPD is an example of such an E3 ligase complex. Selectivity of ubiquitination and recognition of substrates are largely mediated by E3s, either alone or in combination with its bound ubiquitin conjugating enzymes (E2) (Weissman 2001; Themistokleous et al. 2023; Patel et al. 2022).

Defects in ubiquitination and proteasomal protein handling are common features in PD and other chronic neurodegenerative diseases (Ciechanover and Brundin 2003; Dawson and Dawson 2003a; Ding et al. 2017). Consistent with this is the accumulation of a wide spectrum of ubiquitinated protein aggregates in brains of PD patients, such as tyrosine hydroxylase (TH), synphilin-1, α-synuclein, and phosphorylated tau (Liani et al. 2004; Meredith et al. 2004; Zhang and Goodlett 2004), which constitutes the most common form of the idiopathic and genetic disease. In addition to cytoplasmic inclusions, further clues associating a defective UPS with PD pathogenesis came from identification of genetic mutations related to FPD. Of the numerous genes linked to PD (Dawson and Dawson 2003b), two of the genetic mutations, parkin and UCHL1, have direct association with the UPS. This suggests a common pathogenic base in both idiopathic and genetic forms of the disease. A large-scale transcriptomic study conducted in post-mortem human SNpc revealed a decreased expression of *SKP1A*, member of the SCF (Skp1, Cullin 1, F-box protein) (E3) ligase complex specifically in the SN of sporadic Parkinsonian patients (Fig. 1) (Grunblatt et al. 2004; Delgado-Camprubi et al.^2017^; Lee et al. 2023; Zhong et al. 2022). Skp1 forms a component of the SCF complex, which functions as an E3 ubiquitin ligase for the ubiquitin-mediated proteolysis of cell cycle regulators, at the G_1_/S transition of the cell cycle (Feldman et al. 1997; Zheng et al. 2002; Li et al. 2023). SCF complexes represent one major checkpoint within the G_1_/S transition in mitotic cells (Nakayama et al. 2001; Spruck and Strohmaier 2002). About a decade ago, a number of reports have shown an activated cell cycle phenotype in neurodegenerative diseases with abnormal occurrence of cell cycle proteins at an early phase of disease (Smith et al. 2007). Re-entry of quiescent, terminally differentiated neurons, into the cell cycle may result in a mitotic catastrophe and cell death (Copani et al. 2001). For entry into the cell cycle, quiescent neurons of the adult brain must first exit G_0_ and enter the G_1_ phase of the cell cycle. Multiple cell-cycle proteins regulate progression through G_1_, the most important being the products of retinoblastoma (Rb) tumor suppressor and E2F gene families (Weinberg 1995). Progression through G1 phase and entry into S phase is regulated by the activation of G_1_ phase cyclin-dependent kinases (CDKs), namely CDK2, CDK4, and CDK6. CDKs’ activation is regulated by specific phosphorylation and dephosphorylation events, and by binding to cyclins, D-type cyclins in the case of CDK4 and CDK6, and cyclin E in the case of CDK2. These complexes phosphorylate and inhibit the retinoblastoma gene product (pRb), releasing E2F transcription factors for induction of genes required for S-phase progression and DNA synthesis (Sherr and Roberts 1999; Rowland et al. 2023).

A particular decreased expression in PD SN of *HSPA8* gene (heat-shock 70-kDa protein 8), encoding Hsc-70 (70 kDa heat-shock cognate protein), was also reported in the aforementioned study (Grunblatt et al. 2004; Rabey et al. 2020; Grunblatt et al. 2004). Hsc-70 is a member of the Hsp70 family whose expression was severely reduced in PD SN. Hsc-70 acts as a chaperone responsible for recognition of unfolded or aberrant proteins and delivery to a co-chaperone, E3 ligase enzyme CHIP (carboxyl terminus of Hsc70-interacting protein), which, in turn, can cooperate with Hsp90 and/or Hsp70/Hsc-70 and ubiquitinate their attached misfolded substrates (Murata et al. 2003).

The findings that *SKP1A* is decreased in SNpc from PD patients and that humans express only one functional Skp1 isoform (Semple 2003; Mandel et al. 2012a and b; Van Noda et al. 2022; Lee et al. 2021 and 2023) may contribute to a wide impairment in the function of an entire repertoire of proteins, leading to inappropriate expression or activation of cell-cycle players and eventually cell death (Copani et al. 2001). In addition, the decreased activity of Skp1 may affect neuron viability because of a newly suggested structural role of Skp1 not related to its function as an SCF E3 ubiquitin ligase. In this respect, three Skp1-containing, non SCF complexes were described in budding yeast (Hermand 2006). The F-box protein Rcy1 which is required for recycling of the Soluble NSF Attachment protein REceptor (SNARE) Snc1 from endosomes to the plasma membrane constitutes one example of association between an F-box protein and Skp1 independently of SCF (Galan et al. 2001; Wiederkehr et al. 2000). Snc1 encodes homologs of vertebrate synaptic vesicle-associated membrane proteins, also known as synaptobrevin, which mediate the targeting and transport of secretary proteins (Protopopov et al. 1993).

### Gene expression profiling of SPD SN

The heterogenic nature of PD regarding age of onset, symptoms, clinical manifestation, and pathology reflects most probably the involvement of multiple genes, pathways, and downstream effectors rather than single events (Mandel et al. 2003). Thus, a better understanding of the disease etio-pathophysiology is warranted to be reflected in better animal models.

**Fig. 2:**
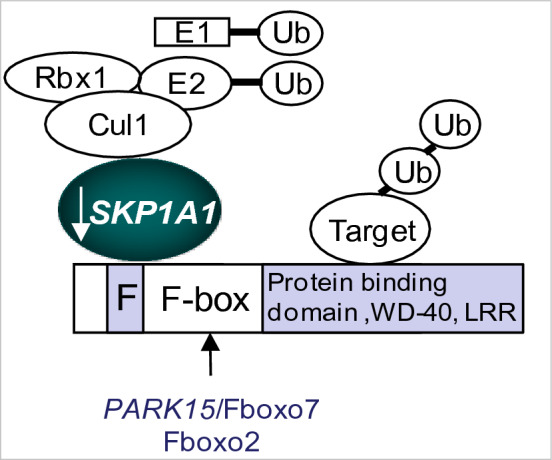
Schematic diagram of ubiquitin E3-SCF complex and its implication in SPD. SCF complexes are modular: the scaffold protein Cul1 (cullin1) interacts at the N terminus with the adaptor subunit Skp1 and at the C terminus with the RING-finger protein Rbx1 that recruits a specific ubiquitin-activating enzyme (E2) for ubiquitylation. The repertoire of proteins processed and degraded by the UPS is amplified. Skp1 decreased expression in the SNpc of PD may impair the assembly with Fbxo7/other F-box proteins and their function in DAergic neurotransmission [Adapted from (Mandel et al. 2009)]

The identification of mutations linked to FPD and the implementation of microarray-based gene expression profiling during the past decade have provided additional clues on how the disease initiates and progresses as well as potential molecular targets that may be of relevance to both familial and sporadic PD (Grunblatt et al. 2004; Hauser et al. 2005; Miller et al. 2006; Moran et al. 2006; Zhang et al. 2005; Gasser 2009). Our earlier gene expression study conducted in post-mortem SN obtained from SPD patients identified a cluster of genes that were most differentially expressed in the SN, compared to non-disease controls (Grunblatt et al. 2004). The transcripts were mainly related to DA transmission and metabolism, and protein handling/degradation mechanisms previously known to be involved in the pathophysiology of the disease. One candidate whose gene and protein expression was robustly decreased in SNpc neurons is Skp1. Skp1 takes part in the Skp1, Cullin 1, F-box protein (SCF) complex, the largest known class of sophisticated ubiquitin–proteasome/E3-ligases, acting in a module-like manner: Skp1 can interact with several F-box proteins, which play an indispensable role in the selection of target proteins for degradation, because each distinct F-box protein usually binds a protein substrate(s) with a degree of selectivity for ubiquitylation through C-terminal protein–protein interaction domains (Fig. 2) (Zheng et al. 2002). This provides functional diversity and increases the repertoire of proteins processed by this complex. Most of the substrates are phospho-modified but also may contain prolyl hydroxyl modification. The fact that humans express only one functional Skp1 isoform (Semple 2003) combined with the decreased expression in PD SN may ascribe for a wide impairment in the function of an entire repertoire of proteins implicated in DAergic neurotransmission. However, *SKP1* function in neuronal cells is unknown. Recently, two separate findings provided significant support to the existence of a functional relation between Skp1 and brain degeneration in mammals: (1) The identification of parkinsonism-causing mutations in *PARK15*/*FBXO7* (Di Fonzo et al. 2009; Randle and Laman 2017; Wang et al. 2021), a Skp1-interacting protein belonging to the family subgroup of F-box domain only (Fbxo) proteins (Dabool et al. 2020). The mutations have been found to cause autosomal recessive early onset parkinsonism. Fbxo7 physically interacts with Skp1 for assembling into an SCF-E3 ubiquitin ligase, and is involved in crucial processes such as apoptosis (Chang et al. 2006; Spagnol et al. 2021). (2) Mice null for another Fbxo subgroup protein, Fbxo2, a brain-enriched F-box protein that associates directly with Skp1, showed a concomitant loss of Skp1 specifically in the cochleae organ accompanied by cellular degeneration and age-related hearing loss (Nelson et al. 2007). Moreover, this and cell culture studies have shown a preference of Fbxo2 to homodimerize or heterodimerize with Skp1 rather than in the traditional SCF tetramer, suggesting an additional role to its acknowledged E3-ligase (Yoshida et al. 2007). Although Skp1 role in neurons is not known, the above findings suggest that a disruption in the function of Skp1 and its interacting molecules may have an impact in PD etio-pathology (Lauterbach 2013; Larsen and Bendixen 2012).

In addition to *SKP1A* decline, which may cause evasion of proteins subjected to SCF/26S proteasome complex degradation, the reduction in the expression of *HSPA8* (coding for Hsc-70), responsible for recognizing unfolded or aberrant proteins, may exacerbate the accumulation of a wide spectrum of ubiquitinated protein aggregates in PD brains such as TH, synphilin-1, α-synuclein, and phosphorylated tau (Murata et al. 2003; Imai et al. 2002; Dabool et al. 2020; Deng et al. 2013). Additional affected functional classes corresponded to DAergic transmission/metabolism [i.e., cyclic ARPP-21, solute carrier family 18 (*VMAT2*), alcohol dehydrogenase 5 (*ADH5*), and *ALDH1A1* (Fig. 1)]. Aldh1 was found to be expressed highly and specifically in DA cells of the SN and ventral tegmental area (VTA) and to be markedly reduced in SNpc DAergic neurons but not in those of the VTA of PD brains (Galter et al. 2003). This is in line with the observation of striatal aldehyde dehydrogenase activity in 6-OHDA-treated rats (Agid et al. 1973) or in cats after electrical-induced lesion (Duncan et al. 1972) which was significantly reduced. Aldehyde derivatives of DA metabolism are highly neurotoxic (Hjelle and Petersen 1983) and Aldh1 is the key enzyme for their metabolism to inert acidic metabolites (Mardh and Vallee 1986). The reduction in gene expression of *ALDH1A1*, *ARPP-21,* and *VMAT2*, which are located within DA-containing neurons of SNpc, may contribute to a failure in DA transmission and metabolism.

On the other hand, increased dysregulation of extracellular matrix cytoskeleton components and iron-oxygen sensor, egl nine homolog 1 (C. elegans) (*EGLN1*), hypoxia-inducible factor-1 prolyl-4-hydroxylase) were observed. The abnormal up-regulation of the *EGLN1* gene in PD brains, recently described as a proline hydroxylase-2 (*PHD2*) belonging to the iron- and 2-oxoglutarate-dependent dioxygenase superfamily (Epstein et al. 2001), may exacerbate the OS status and promote iron-induced aggregation of α-synuclein. *PHD2* activation also results in proteasomal degradation of hypoxia-inducible factor (HIF) and iron regulatory protein 2 (Irp2), with subsequent decreases in cell survival/proliferation, glucose, and iron metabolism genes (Hanson et al. 2003; Wang et al. 2004). Our gene expression profiling study is supported by recent microarray studies from independent laboratories conducted also in human Parkinsonian SN, showing dysregulation of similar functional classes (Grunblatt et al. 2004; Hauser et al. 2005; Miller et al. 2006; Moran et al. 2006; Zhang et al. 2005; Gasser 2009).

### Skp1 content is reduced in in vivo and in vitro models of PD

**Fig. 3:**
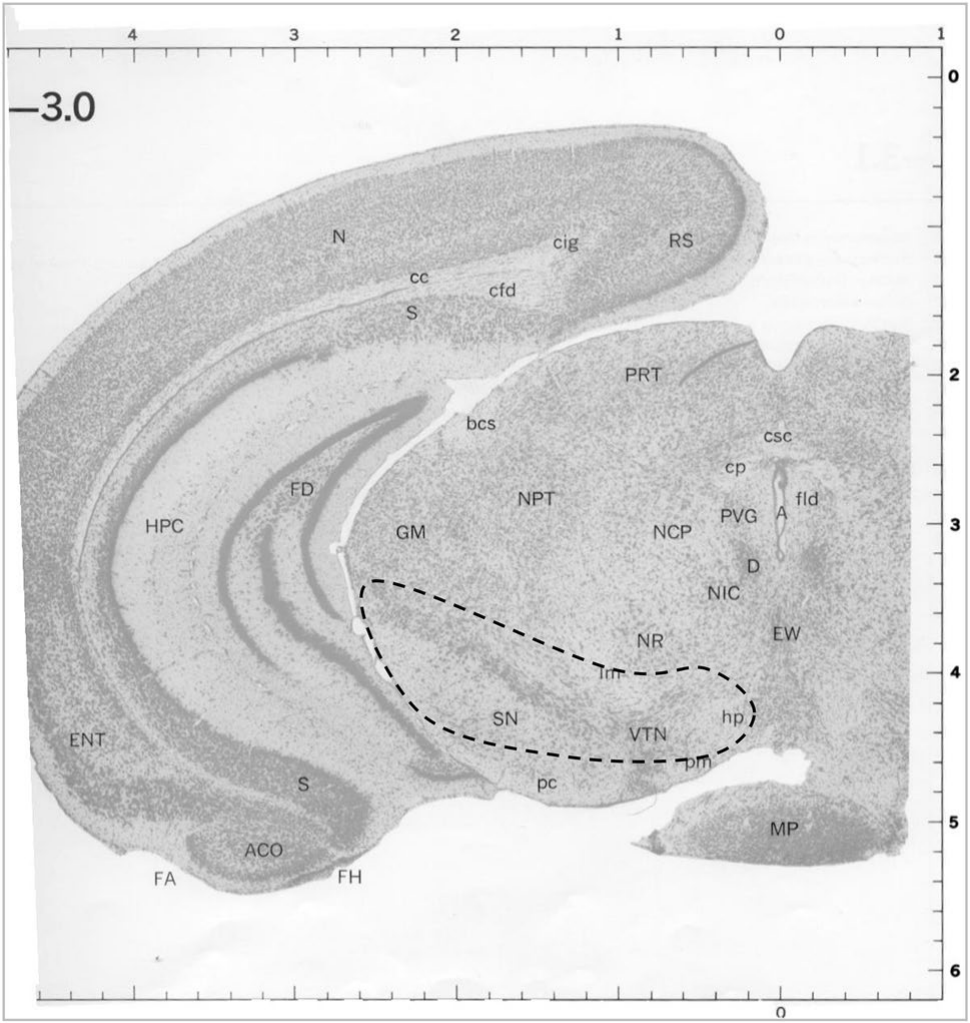
Midbrain micro-puncture

Our large-scale transcriptomics and confirmatory proteomic analyses in human post-mortem tissues have shown that* SKP1A* gene and protein are significantly reduced in the SNpc of PD patients, compared to age- and gender-matched controls without neurological disorders (Grunblatt et al. 2004). This was accompanied by parallel reductions in *DA*T, *VMAT2*, *HSPA8*, and *ALDH1A1*, playing central roles in the processing of aberrant proteins, regulation of DA disposition in the synapse and cytosol, and degradation of toxic aldehyde derivatives of DA. To get an insight into the role of Skp1 in DAergic neurodegeneration, we followed its expression in “in vitro” and “in vivo” models of PD, and the corresponding expression of DAergic-related phenotype markers (Fig. 3).

#### Skp1 protein is rapidly down-regulated by MPP^+^ in *SN4741* cells and decreases in the midbrain of MPTP-induced parkinsonian mice

**Fig. 4:**
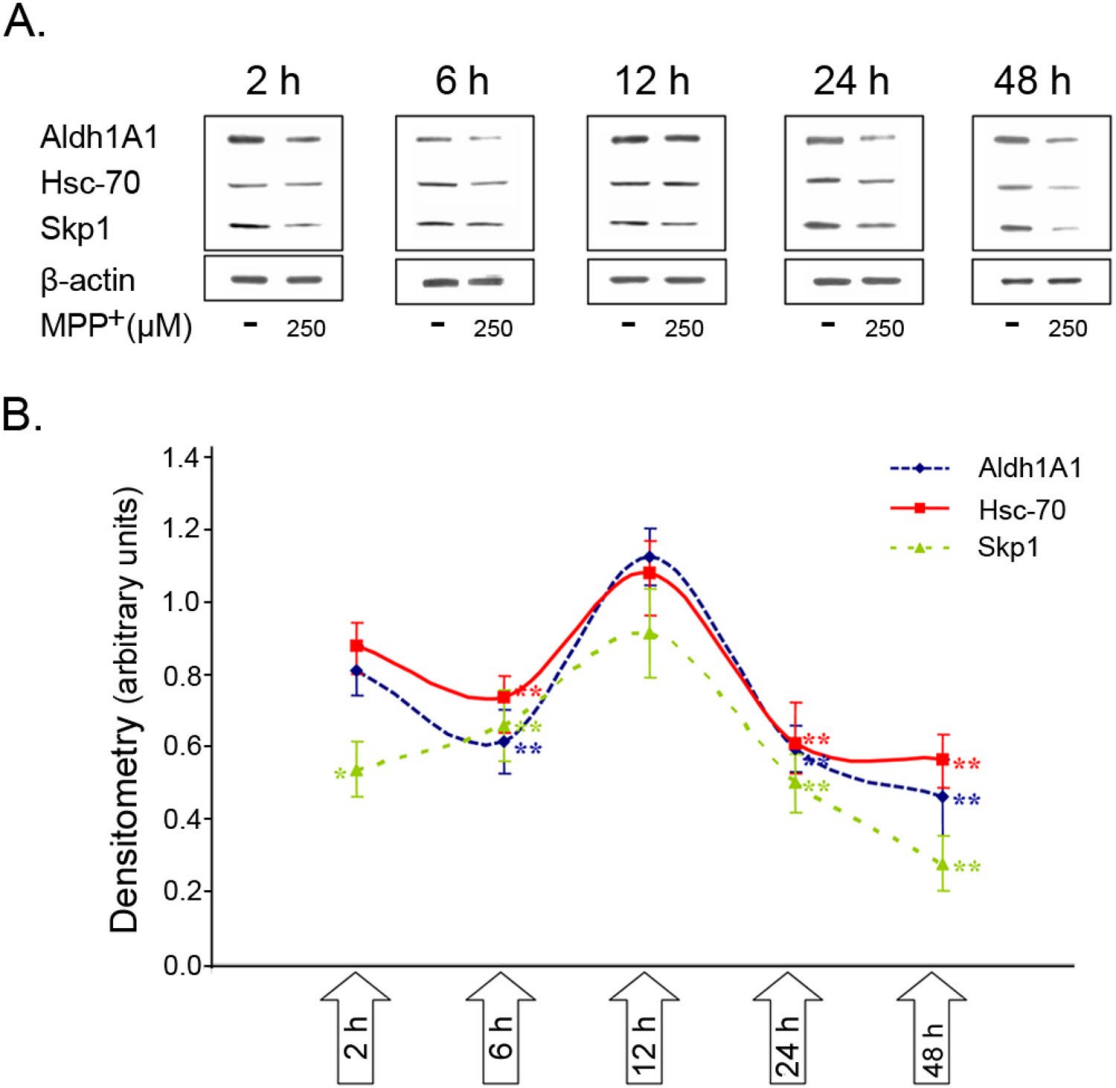
Effect of MPP^+^ on expression levels of Skp1, Aldh1, and Hsc-70 proteins. Mouse SN4741 cells were seeded in 10% FCS containing medium for 24 h, then fresh medium-containing 0.5% FCS and MPP^+^ (250 µM) was added, and cells were harvested at increasing time intervals: 2 h, 6 h, 12 h, 24 h, and 48 h. Following termination of incubation, cells were lysed in RIPA buffer. **A** Representative gel micrographs of protein levels evaluated by Western blot analysis using specific antibodies. **B** Bands were quantified by densitometry and normalized to β-actin. The results are the mean ± SEM of three independent experiments conducted in 2–4 replicates. *p < 0.05, **p < 0.01 vs. control

We first conducted a time-course study with naïve *SN4741* DAergic cells exposed to MPP^+^ (250 μM) for various time intervals and the expression of Skp1 protein was monitored by western blotting (Fig. 4A). MPP^+^ caused an acute (2 h) down-regulation of Skp1 (54.0 ± 7.6 of control), returning to control values 12 h later and a long-term (48 h), more pronounced reduction (to 28.0 ± 7.6% of control) (Fig. 4B). Similarly, the expression pattern of the DAergic marker Aldh1 and the chaperone Hsc-70, a member of the Hsp70 family followed that of Skp1 (at 48 h: 46.3 ± 10.5% and 56.7 ± 7.0% of control, respectively).

**Fig. 5:**
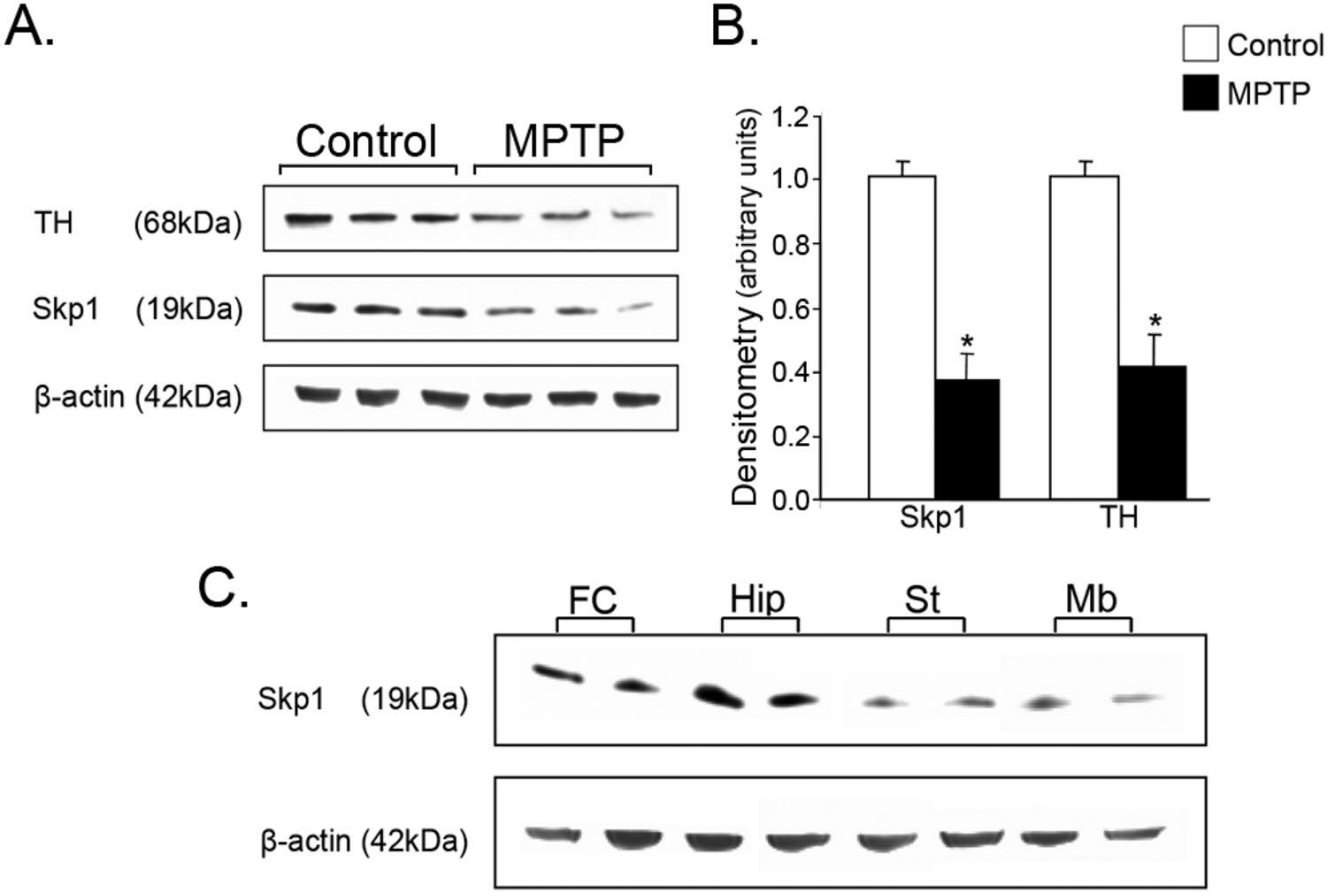
Expression of Skp1 protein in MPTP-treated mouse midbrain and its abundance in brain tissue. C57/Bl mice (*n* = 6–9) were treated with the parkinsonism-inducing neurotoxin, MPTP (20 mg/kg/day) for 4 days followed by additional 4 day resting period. **A** and **B** Mice midbrains homogenates were resolved by SDS-PAGE and immunoblotted with specific rabbit anti-Skp1 and mouse anti-TH antibodies. Representative blot images are shown. Analysis of the bands, given in arbitrary units, is represented graphically. The values are the mean ± SEM from two independent experiments. *p < 0.01 vs. MPTP. **C** The relative abundance of Skp1 protein in mouse brain from naïve, untreated mice (*n* = 5) was assessed by Western analysis in tissue lysates from different brain areas. *FC* frontal cortex, *Hip* hippocampus, *St* striatum, *Mb* midbrain

We next sought to extend these studies to an animal model of PD, employing the neurotoxin MPTP, the parental generator of MPP^+^ and measuring the levels of Skp1 in the midbrain that contains the DA producing neurons of the SNpc. The protein level of Skp1 was significantly decreased (37.3 ± 8.1%) in the midbrain of MPTP-injured mice compared to vehicle-treated mice, further supporting the assumption of a crucial role of Skp1 in DAergic function (Fig. 5A and B). A parallel decline was seen in TH protein content. To assess the relative abundance of Skp1 in the brain, protein lysates from different brain areas of naïve mice were electrophoresed and blotted with an antibody against Skp1. Figure 5C reveals that the midbrain and striatum contain the lowest Skp1 protein levels, compared to frontal cortex, hippocampus, and cerebellum.

#### Enforced expression of wild-type *SKP1A* protects against MPP^+^ toxicity and proteasomal inhibition

**Fig. 6:**
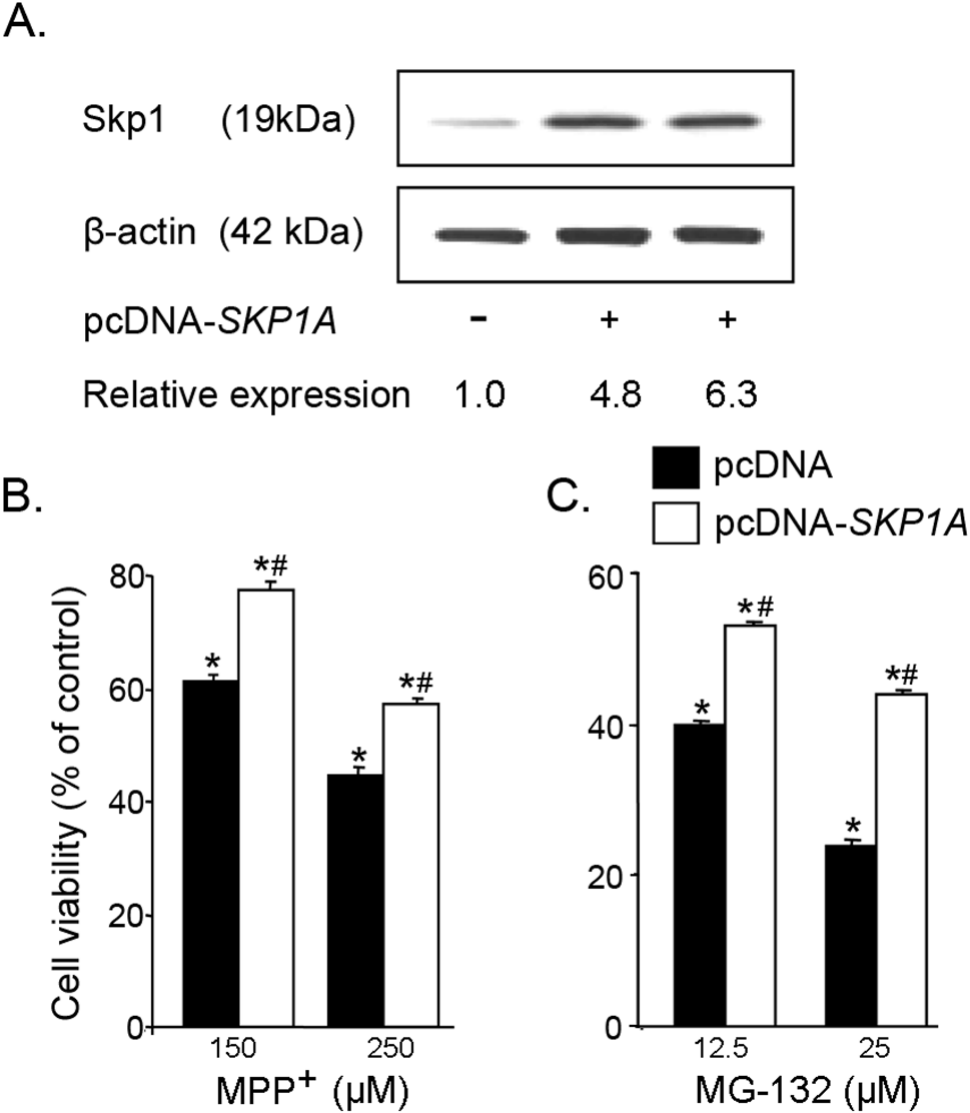
SKP1A overexpression decreases the susceptibility to MPP^+^ and proteasomal inhibition injury. SN4741 cells were stably transfected with expression vector pcDNA3.1hygro-SKP1A or empty expression vector, pcDNA (selection with Hygromycin). **A** Representative gel micrograph of Skp1 protein levels evaluated by Western blot analysis. The values of Skp1 were normalized to β-actin and expressed as relative expression of control (pcDNA3.1_hygro_ vector). Transfected cells were injured with (**B**) MPP^+^ (150 or 250 µM) for 48 h or with (**C**) the proteasome inhibitor MG-132 (12.5 and 25 µM, dissolved in DMSO) for 6 h. Cell viability was assessed by the MTT test. The results are mean ± SEM of 3–5 independent experiments and expressed as percentage of control (pcDNA3.1_hygro_ empty vector). *p < 0.01 vs. empty vector; #*p* < 0.01 vs respective control (without MPP.^+^ or MG-132)

The assumption that Skp1 plays a defensive role (Dabool et al. 2020) was further examined in *SN4741* cells stably transfected with an expression vector carrying the mouse *SKP1A* gene coding sequence (*pcDNA3.1*_*hygro*_-*SKP1A*) and damaged with MPP^+^. As shown in Fig. 6A, the protein expression level of Skp1 was increased up to 6.3-fold over the control level (cells transfected with *pcDNA3.1*_*hygro*_-empty vector). The overexpression of Skp1 protein was found to protect the DAergic cells against damage induced by exposure to MPP^+^ (150 and 250 µM) for 48 h (Fig. 6B) (survival was increased from 61.1 ± 1.4% to 77.5 ± 1.5% at 150 µM and from 44.3 ± 1.8% to 57.1 ± 1.3% at 250 µM). A more robust protection was afforded in cells injured by pharmacological inhibition of the proteasome. Figure 6C shows that neuronal cultures transfected with Skp1 duplicated the survival index of naïve *SN4741* exposed for 6 h to the reversible proteasome inhibitor, peptide aldehyde MG-132 (12.5 and 25 µM) (from 39.6 ± 0.7% to 52.9 ± 0.6% and from 23.9 ± 0.8% to 43.9 ± 0.4%, respective to each concentration), compared to empty vector-transfected cells. Thus, Skp1 overexpression can protect DA neurons from proteasome inhibition- and toxin-mediated cell damage (Delgado-Camprubi et al. 2017).

### Deficiency of *SKP1A* impairs DAergic neuron phenotype

#### RNAi-mediated silencing of *SKP1A* in *SN4741* cell line gene and protein expression

As stated in the “research aims” (see the section “Discussion”), our second objective was to study whether the deficiency of *SKP1A* closely recapitulates, at the cellular level (in vitro), cardinal features of the neuropathology occurring in DA neuron degeneration, including an increase susceptibility to cell death and a decline in the expression of DAergic phenotypic markers. The clonal SN DA cell line *SN4741* was selected for this purpose, since it contains an abundant source of homogeneous DA neurons that could be efficiently infected and the phenotype maintained along generations. *SN4741* cells are derived from progenitor mesencephalic cells from transgenic mouse embryos arrested at an early DA developmental stage and express high levels of neuronal markers, neurotrophins, and receptors (Son et al. 1999), maintaining many of the characteristic features of SN DA neurons.

It is acknowledged that the slowly progressive loss of DAergic neurons reflects initial alterations in particular biological processes and their genes, occurring at early stage in PD which propagate over the course of the years (Mandel et al. 2007). Thus, the idea was to gradually develop phenotypic alterations of the neurons by *SKP1A* silencing but not ablation, to better emulate the neurodegenerative process. For gene knocking down purposes, we have chosen the LV-mediated RNAi approach. RNAi is a potent tool to suppress genes in human cells and is considered a potential therapeutic approach in neurological disorders. The signals that trigger RNAi are small double-stranded RNA (dsRNA) molecules which are recognized and cleaved by Dicer, a member of the RNaseIII family of dsRNA-specific endonucleases, generating the so-called siRNAs. The siRNAs are incorporated into the RNAi silencing complex (RISC), guiding the RISC to complementary mRNA, and thereby triggering gene-specific mRNA degradation (Meister and Tuschl 2004; Filipowicz et al. 2005; Tomari and Zamore 2005).

**Fig. 7:**
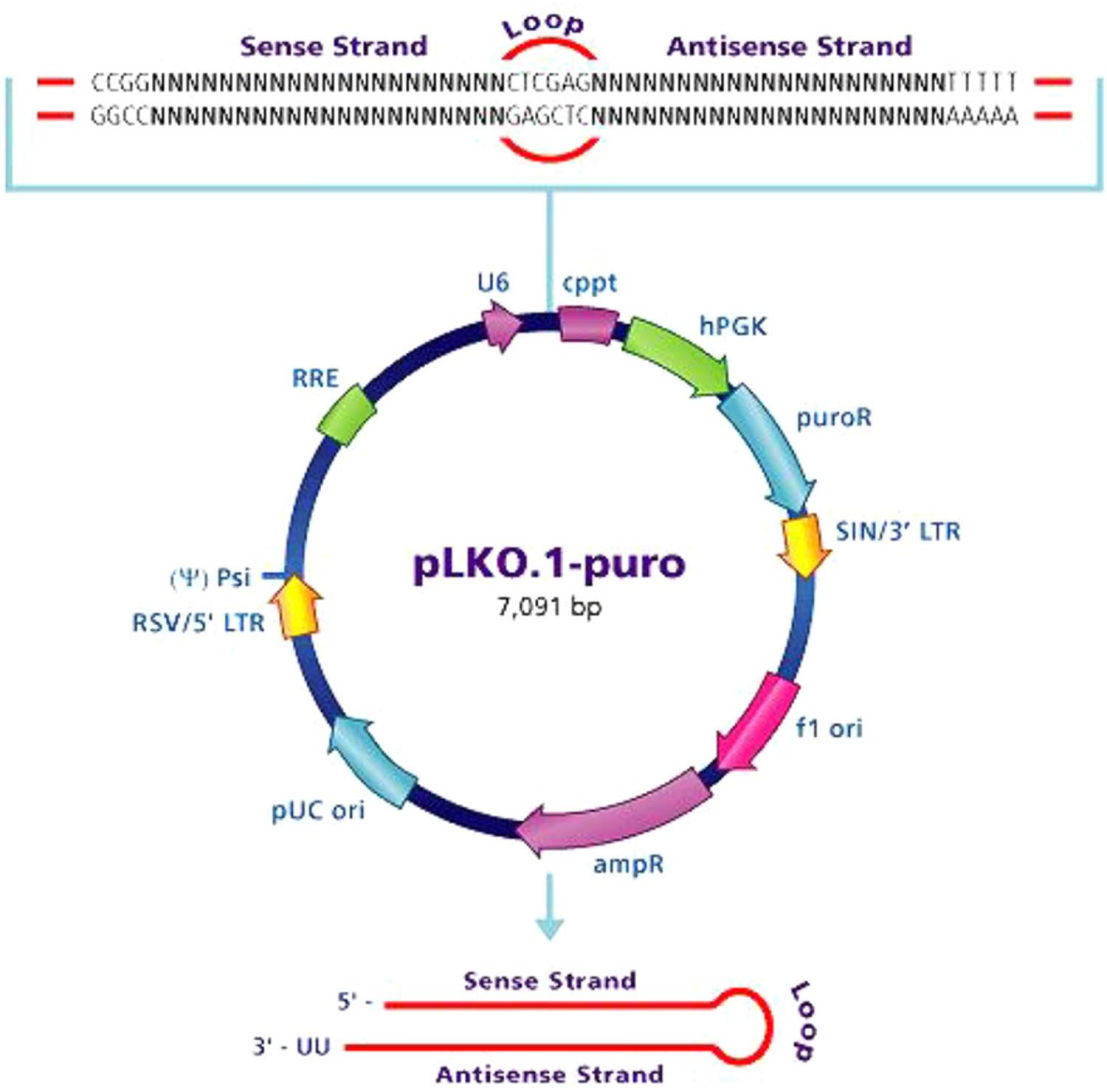
pLKO.1-puro vector (Sigma). This vector allows production of lentiviral particles. Stable gene silencing is selected using the puromycin selectable marker, while self-inactivating replication incompetent viral particles can be produced in packaging cells (HEK293T) by co-transfection with compatible packaging plasmids. Lentiviral-based particles permit efficient infection and integration of the specific shRNA construct into differentiated and non-dividing cells, such as neurons, overcoming low transfection, and integration difficulties when using these cell lines. Compared to siRNA and other vector-based systems, pLKO.1 provides solutions for long-term knockdown and phenotypic observation, transduction of difficult or sensitive cell lines (non-dividing cells or primary cells), and is an economical renewable resource

**Fig. 8:**
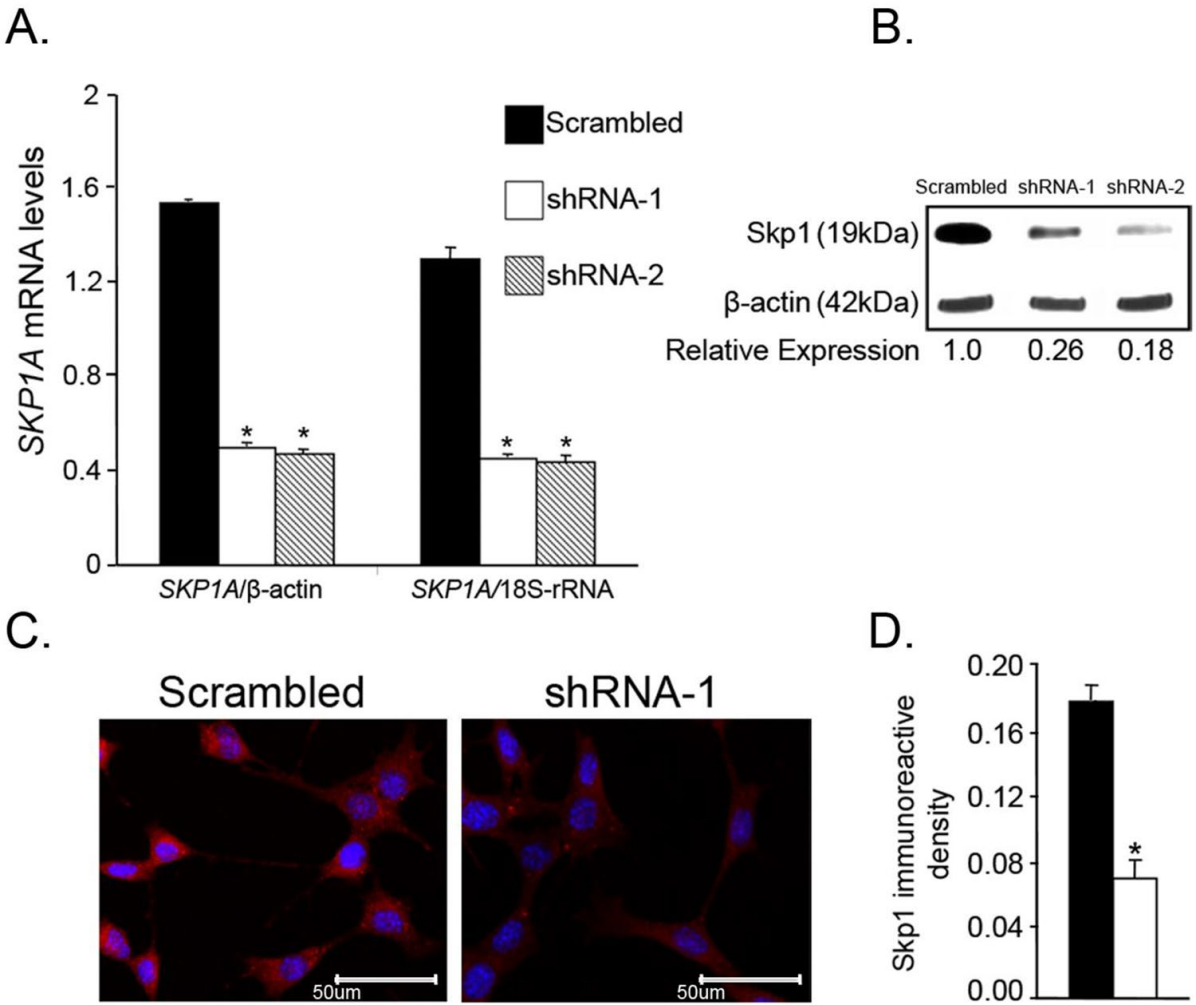
RNAi-mediated silencing of SKP1A in SN4741 cell line. **A** SN4741 cells were infected for 1 week with LVs’ plasmid vectors encoding shRNAs targeting SKP1A, shRNA-1, and shRNA-2 or a scrambled sequence. RNA was extracted and converted to single stranded for analyzing by Q-PCR. The relative expression level was assessed by normalizing to the housekeeping genes 18S-rRNA and β-ACTIN. Values are mean ± SEM from two independent experiments conducted in 2–5 replicates. *p < 0.01 vs control (scrambled). **B** After lentiviral infection cells, homogenates were analyzed by Western blotting using Skp1-specific antibody. Bands were quantified by densitometry and normalized to β-actin. **C** and **D** Scrambled or shRNA-1 infected cells were grown with full serum (FS) support at 33 °C and after fixation and permeabilization, Skp1 protein was detected by fluorescence microsentative fields from two independent experiments. Chart represents mean immunoreactive density of 6–9 separate fields from 2 independent experiments normalized to number of cells in each field. *p < 0.01 vs. control (scrambled)

Initially, we infected *SN4741* cells with short hairpin RNA lentiviruses (shRNA-LVs) encoding the murine transcript of the *SKP1A* gene, a member of the SCF (E3) ligase complex, to the cells. shRNA encodes two perfectly complementary short RNA strands linked by a short loop (see Fig. 7) and typically is transcribed from DNA polymerase III (pol-III) promoters, hence mimicking the siRNA pathway even in a more significant potent way (Amarzguioui et al. 2005). LVs are members of the retrovirus family derived from human immunodeficiency virus type 1 (HIV-1) that have acquired additional properties, including a unique ability to translocate across the nuclear membrane and infect mitotic and post-mitotic cells (Naldini et al. 1996). Because of the toxicity of some viral components, LV particles have been produced with packaging plasmids encoding the envelope and RNA encapsidation proteins together with the *pLKO.1-puro* shRNA plasmid (Fig. 7). The advantage of using this technique is that the LVs stably integrate the DNA copy of their genome into the host chromosome during their replication cycle and therefore remains stable for many generations with a long-term and sustained effect. Two different sequences of shRNA-encoding LVs targeting the transcript of murine *SKP1A* were assayed to endogenously generate siRNA-mediated silencing of *SKP1A* (designated shRNA-1 and shRNA-2). Q-PCR analysis revealed a significant decrease in mRNA of *SKP1A* (~ 70–80% reduction) compared to scrambled shRNA-infected cells (Fig. 8A). The scrambled shRNA negative control activates RISC and the RNAi pathway, but does not target any human or mouse genes. A similar down-regulation of *SKP1A* mRNA by the two shRNA vectors was obtained when Q-PCR values were normalized to either β-*ACTIN* or *18S-rRNA*. Confirmatory western (Fig. 8B) and immunofluorescence labeling (Fig. 8C, D) of Skp1 protein showed a ~ 75% and ~ 60% reduction in the expression levels of Skp1 protein in cells infected with both LVs or with shRNA-1, respectively.

#### *SKP1A* silencing induces cell morphology and cell-cycle alteration

**Fig. 9:**
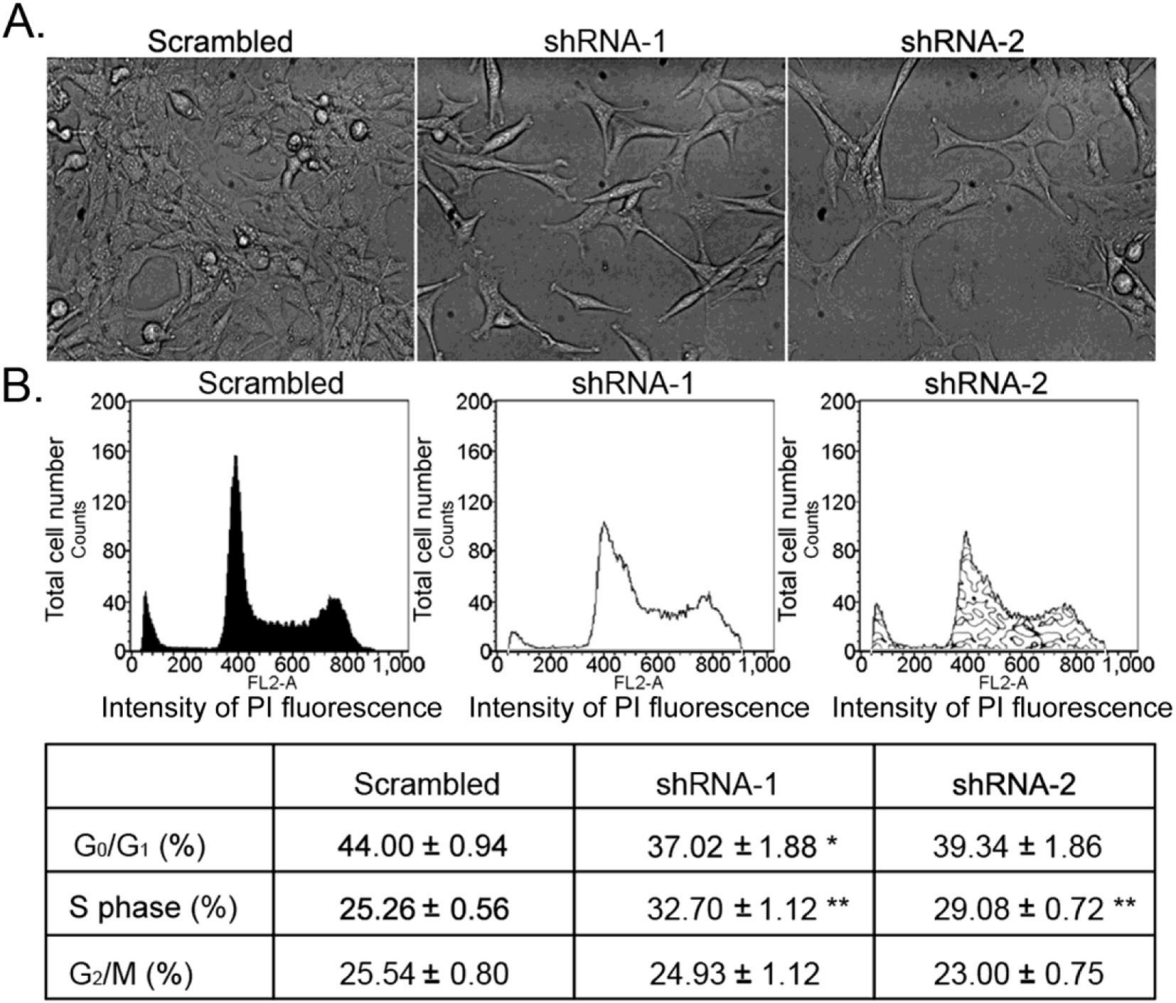
Effect of SKP1A silencing on cell cycle progression. **A** Lentiviral-infected SN4741 cells were seeded in DMEM medium with 10% FCS and puromycin for selection. SKP1A-infected cells appear less dense than their corresponding control (scrambled vector). Pictures were acquired after 24 h, using an inverted microscope connected to a digital camera (20 × objective). **B** shRNA-1, shRNA-2, and scrambled vector infected cells were gently suspended in a hypotonic fluorochrome solution, incubated in the dark at 37 °C for 30 min, and analyzed for DNA content on a logarithmic scale by FACS calibur flow cytometer with Cell Quest research software; 3X10^4^ events per sample were acquired. Both shRNAs decreased the number of cells in G_0_/G_1_ and increased the number of cells in S phase, with a delay in completion the cell cycle. Values are mean ± SEM from three independent experiments conducted in three replicates. *p < 0.05, **p < 0.01 vs. control scrambled vector infected cells

*SKP1A* silenced cells display distinctive phenotypic characteristics when compared to the control (Randle and Laman 2017). As shown in Fig. 9A, RNA interference (RNAi)-mediated *SKP1A* inhibition caused neurite extensions and thinner cell bodies, compared with shRNA-scrambled infected cells. Also, *SKP1A-*shRNA-infected cells appear less dense than control, suggesting a defect in cell replication. This result prompted us to initially examine the effect of *SKP1A* gene silencing on cell cycle progression. FACS analysis revealed that infection with either shRNA-1 or shRNA-2 diminished the population of G_0_/G_1_ phase cells from 44.0% (scrambled) to 37.0% and 39.3% respectively, and increased the number of cells in S phase from 25.3% to 32.7% and 29.1%, with a delay in completion the cell cycle (Fig. 9B). The proportion of cells in G_2_/M phase did not change significantly and no increase in the percentage of apoptosis was visible in the *SKP1A*-silenced population compared to control. Furthermore, no visible decrease in cell viability was observed in the course of 72 h in the *SKP1A*-deficient cells grown under permissive conditions: 2% FCS at 33ºC (116 ± 0.4% compared to 24 h culture).

#### RNAi-mediated *SKP1A* inhibition reduces the expression of DAergic markers in *SKP1A*-silenced cells

**Fig. 10:**
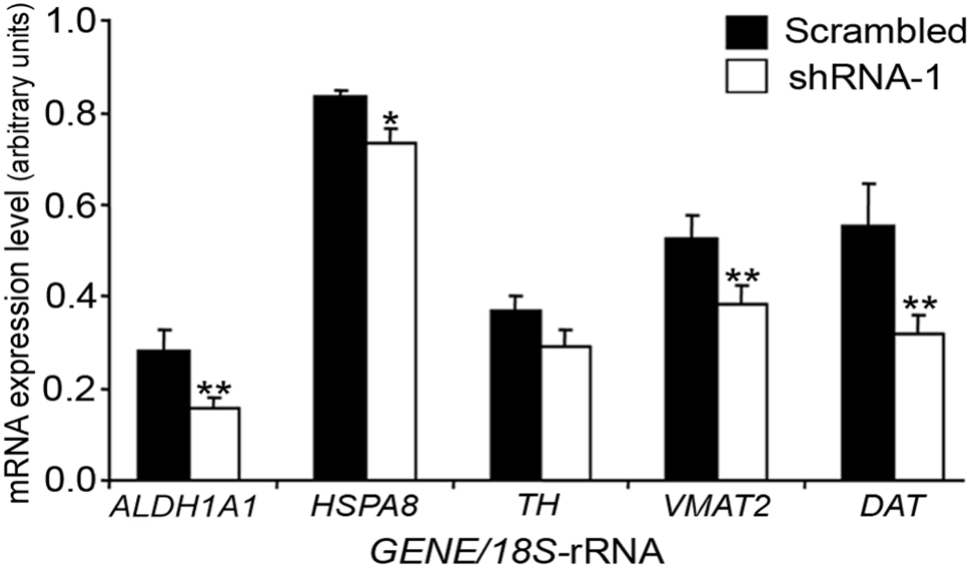
Effect of RNAi-mediated SKP1A inhibition on the expression of DAergic markers. Following infection with SKP1A shRNA-1 or scrambled vector, the mRNA levels of ALDH1A1, HSPA8 (encoding Hsc-70), TH, DAT, and VMAT2 were quantified by Q-PCR. The relative expression was assessed by normalizing to the housekeeping gene 18S-rRNA. Values are mean ± SEM from two independent experiments conducted in 2–5 replicates. *p < 0.05; **p < 0.01 vs. scrambled vector

To further examine the role of Skp1 in DAergic neuron phenotype, we assessed the gene expression of phenotypic markers of DA neurons in Skp1-deficient cells. Given the similar results obtained with the two *SKP1A* shRNA-LVs, shRNA-1 was selected for further studies. Q-PCR analysis showed that *SKP1A* shRNA-1-mediated silencing, caused a significant decline in *DAT* and *VMAT2* mRNA (~ 43% and ~ 27% of scrambled vector, respectively) both being key components in the regulation of DA disposition in the synapse and cytosol. Another gene whose expression was significantly down-regulated (~ 44%) was *ALDH1A1* (Fig. 10). This isoform was found to be expressed highly and specifically in DA cells of the SN and ventral tegmental area (VTA) (Galter et al. 2003) having a role in the neutralization of toxic aldehyde derivatives of DA. The deficiency of Skp1 brought also a slight but significant reduction in *HSPA8*. Similar results were obtained when normalizing the Q-PCR values to *β-ACTIN* (not shown). These findings establish a role for Skp1 in neuronal differentiation.

##### SKP1A is induced upon neuronal differentiation

**Fig. 11:**
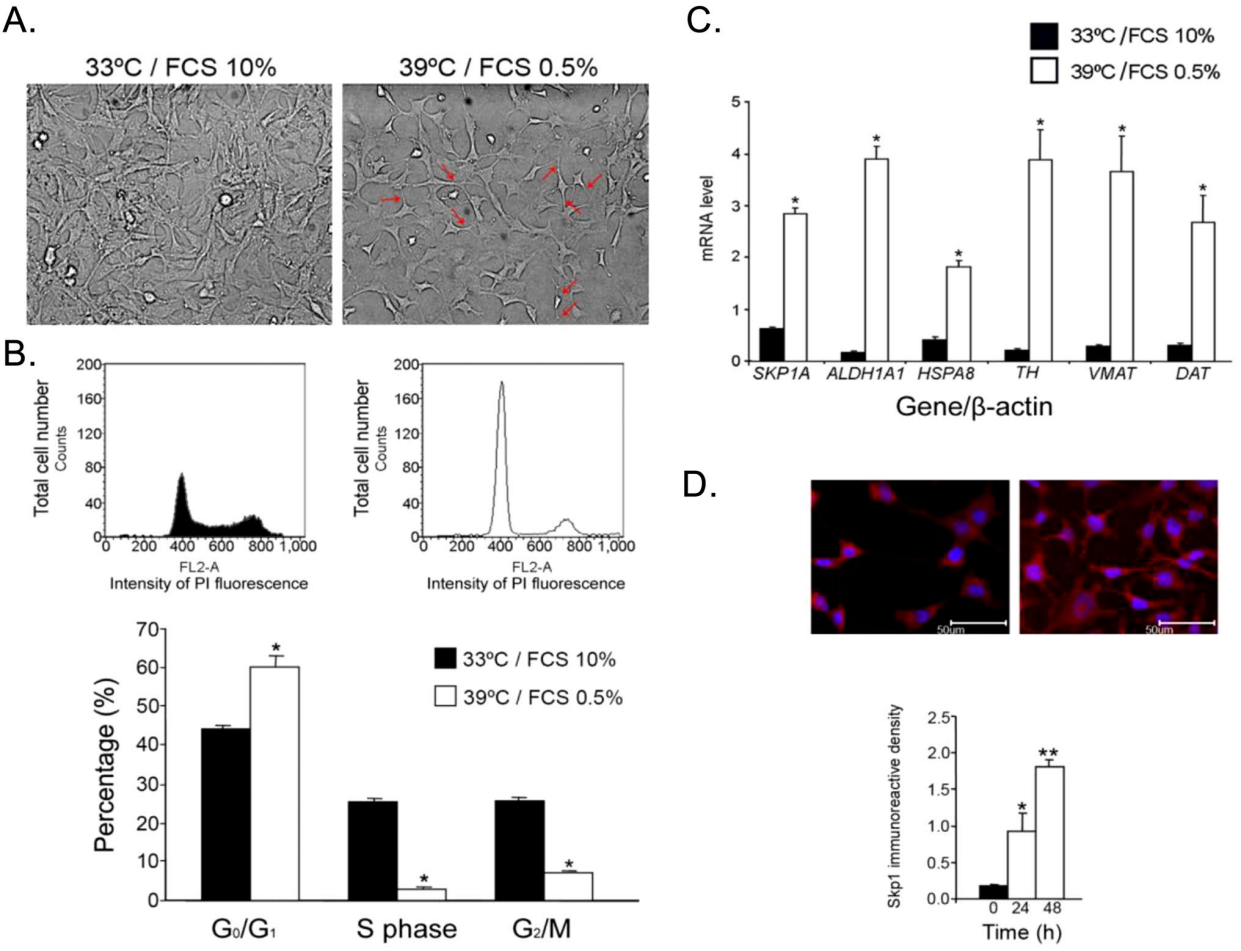
Differentiative features of naïve SN4741 cells. **A** SN4741 cells carrying the temperature-sensitive mutant from the oncogene, SV40Tag-tsA58, have a fibroblast-like flat morphology at 33 °C. Under differentiation conditions (i.e., non-permissive temperature of 39 °C and 0.5% FCS), the SN4741 cell line ceased proliferation and after 48 h, started to display a neuronal morphology with extensive neurite outgrowth. **B** Representative histograms and percentage of cells at different phases of the cell cycle as analyzed by FACS. Values are mean ± SEM from three independent experiments conducted in three replicates. *p < 0.01 vs control (33 °C, FCS 10%). (C) SKP1A and DA neuron-specific markers were analyzed by Q-PCR in naïve SN4741. RNA was extracted from non- and differentiated cells and the expression levels of various DA neuron genes assessed. The relative expression level was assessed by normalizing to β-ACTIN. Values are mean ± SEM from two independent experiments conducted in 2–5 replicates. *p < 0.01 *vs*. *SN4741* cells treated under permissive conditions (FS and 33ºC). **D** After cell fixation and permeabilization Skp1 protein was detected by fluorescence microscopy using a specific Skp1 primary antibody. The chart represents mean immunoreactive density of 6–9 separate fields from 2 independent experiments normalized to number of cells in each field; *p < 0.05, **p < 0.01 *vs*.* SN4741* cells under permissive conditions

The suggested role of Skp1 in neuronal differentiation was reciprocally examined in naïve *SN4741* cells. *SN4741* carry the temperature-sensitive mutant from the oncogene, SV40Tag-tsA58 and proliferate at 33 °C with a doubling time of 36 h, having a fibroblast-like flat morphology with less-prominent neurite growth (Fig. 11A). However, when naïve *SN4741* cells are shifted to the non-permissive temperature of 39 °C and FCS is reduced to 0.5%, they cease to proliferate and, after 48 h, display a neuronal morphology with extensive neurite outgrowth and long bipolar or multipolar processes (Fig. 11A, arrows) (Son et al. 1999). Indeed, FACS analysis showed an increased number of cells in G_0_/G_1_ (60.17 ± 2.66% at the restrictive temperature, compared to 44.0 ± 0.9% at the permissive temperature), while the number of proliferating cells in the S phase and G_2_/M phase dropped considerably from 5.3 ± 0.6% to 2.9 ± 0.3% and from 25.5 ± 0.8% to 7.1 ± 0.4%, respectively (Fig. 11). The indication of a differentiation state was further corroborated by monitoring the gene expression of the DA neuron-specific markers, *DAT*, *TH*, *VMAT2,* and *ALDH1A1*, which, as expected, were significantly up-regulated at the restrictive differentiation temperature (Fig. 11C, values normalized to *β-ACTIN*). Comparable results were obtained when normalizing to *18S-rRNA* (not shown). An important finding is the prominent elevation in both Skp1 gene and protein levels upon cell cycle arrest, as assessed during 48 h of culture by Q-PCR and immunocytochemistry (up to 4.6 ± 0.2 and 10.1 ± 0.6-fold of non-differentiated cells, respectively, Fig. 11C and D). This observation further supports a central role of Skp1 not only on cell survival but also in DAergic differentiation.

#### Skp1 deficiency is lethal to differentiated *SN4741*

**Fig. 12:**
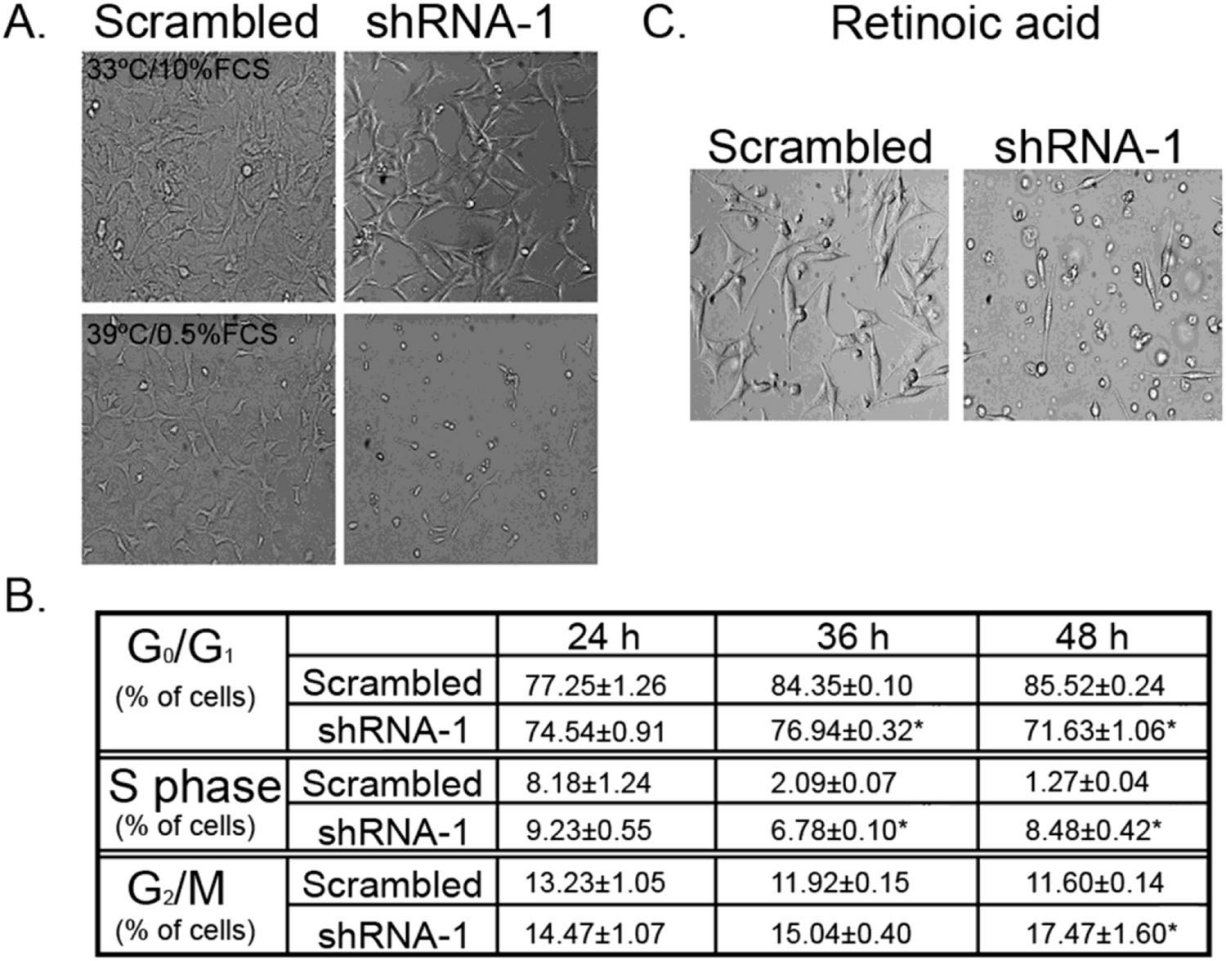
Differentiation-induced lethality of *SKP1A*-silenced cells. **A** *SKP1A* silenced (shRNA-1 infected) and non-silenced cells (scrambled) seeded in 10% FCS containing medium were induced to differentiate at restrictive temperature for up to 48 h. Pictures were acquired using an inverted microscope connected to a digital camera (10 × objective). **B** Cells were analyzed for DNA content by FACS. The percentage of cells in the G_0_/G_1_, S, and G_2_/M fractions was calculated. Values are mean ± SEM from three independent experiments conducted in three replicates. *p < 0.01 *vs*. scrambled. **C **Differentiation with retinoic acid (10 μM)

Since adult neurons in the brain are in a state of terminal differentiation, we sought to examine whether the silencing of *SKP1A* gene would have any phenotypic effect in cells that had exit the cell cycle. To test this hypothesis, *SN4741* cells deficient in *SKP1A* were shifted to the restrictive temperature to induce differentiation and assayed by FACS for a period of 48 h. Given the similar results obtained with the two *SKP1A* shRNA-LVs, shRNA-1 was selected for further studies. A prominent cell mortality was evident as manifested by the appearance of cells with short neurites largely retracted and shrunken cell bodies which accentuated with the duration of culture, compared to scrambled infected cells (Fig. 12A). This was accompanied by a progressive reduction in the percentage of cells in G_0_/G_1_ (Fig. 12B) in parallel to an increase in S phase, indicating an attempt to escape from cell cycle arrest. To rule out any possible contribution of conformational changes in proteins that might have occurred when shifting the cells to 39 °C, differentiation was also induced by adding the well-established regulator of cell growth and differentiation, retinoic acid (10 µM). A similar picture was obtained after daily exposure to retinoic acid (Fig. 12C).

### *SKP1A*-deficiency cause proteinaceous inclusions formation in differentiated *SN4741* cells

**Fig. 13:**
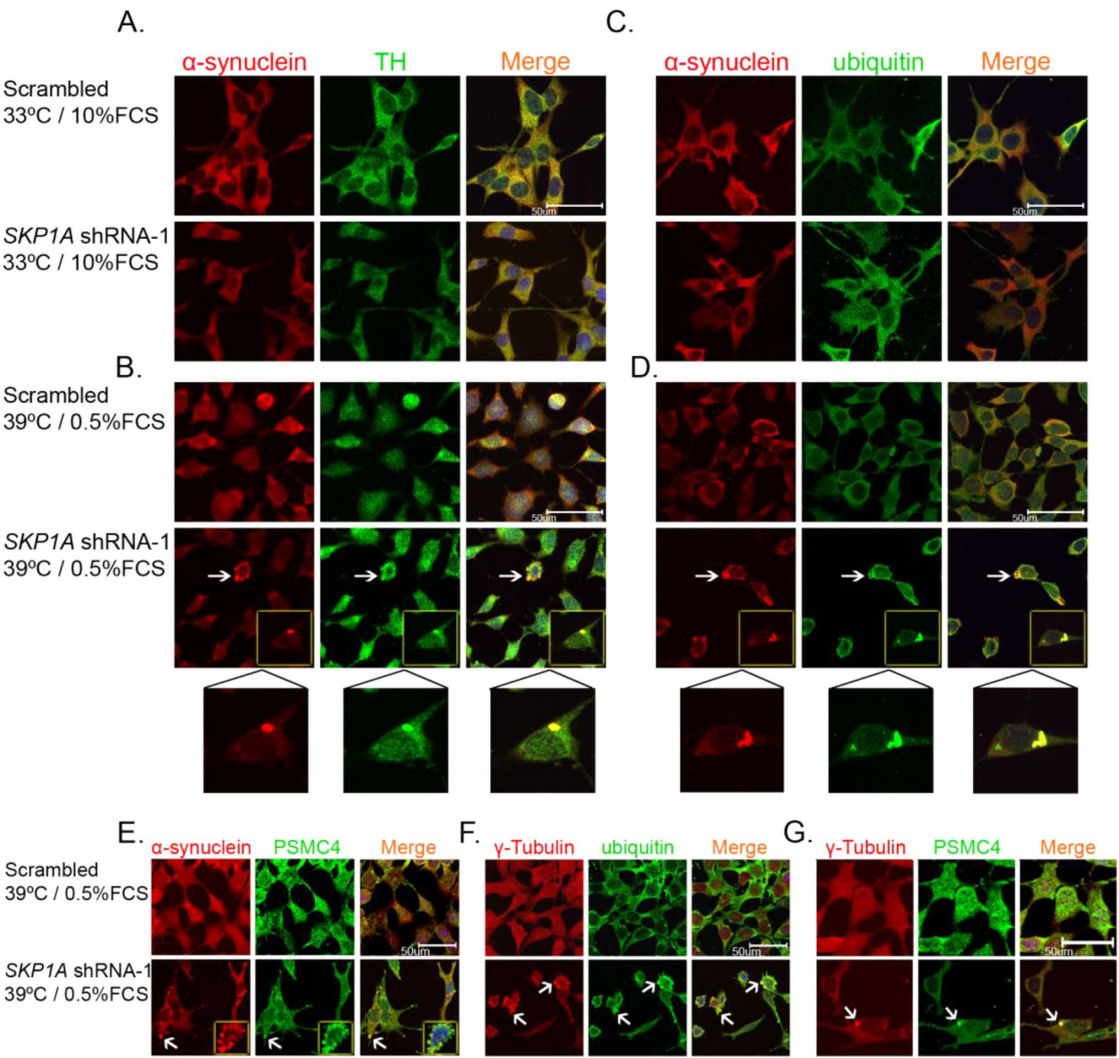
*SKP1A* silencing causes inclusion body formation in differentiating cells. Scrambled and lentiviral-stable infected *SN4741* cells grown on coverslips were induced to differentiate under restrictive conditions for 24 h (pre-lethal stage), fixed, and subjected to double immunostaining with (**A**, **B**) anti-α-synuclein (red) and anti-TH (green) antibodies, (**C**, **D**) anti-α-synuclein (red) and anti-ubiquitin (green) antibodies, **E** anti-α-synuclein (red) and anti-PSMC4 (green) antibodies, **F** anti-γ -tubulin (red) and anti-ubiquitin (green) antibodies, **G** anti-γ-tubulin (red) and anti-PSMC4 (green) antibodies. Scrambled-infected (control) cells show a diffuse staining pattern and do not show any inclusions at both permissive and restrictive temperatures (**A**–**D**, upper images).* SKP1A*-deficient cells developed multiple and perinuclear (**B**, **D**, **E***,* bottom images, see arrows and insets) inclusion bodies 24 h after induction of differentiation, with characteristics of aggresomes, staining for γ-tubulin (**F**, **G**). The reactivity of the aggregates to the different antibodies demonstrated a similar pattern as indicated by co-localized, yellow immunostaining in the overlayed right-hand panel, with the addition of Topro staining (blue) to identify the nucleus. No fluorescence was detected when the primary antibody was omitted. Each panel shows a representative picture of 10–15 views in two independent experiments

**Fig. 14:**
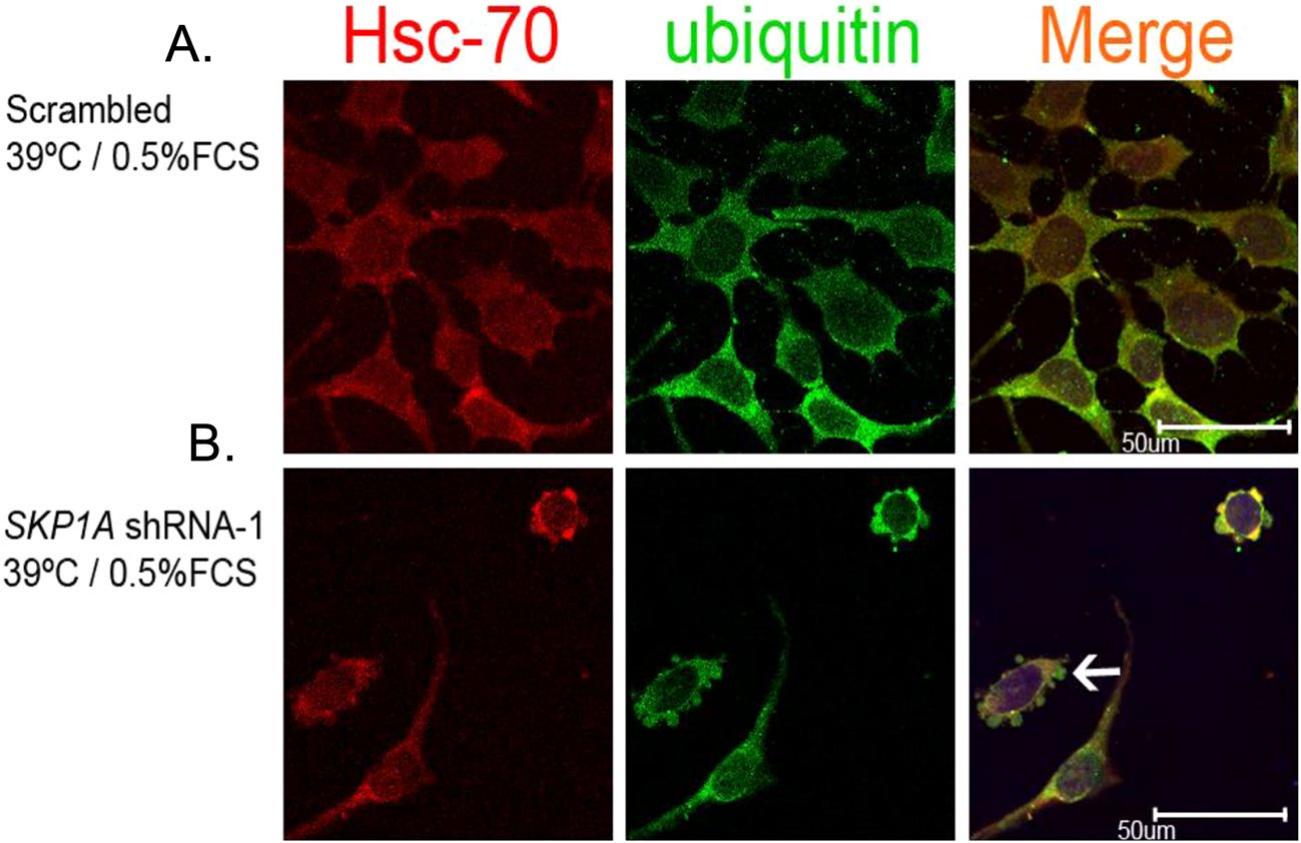
Ubiquitin colocalizes with Hsc-70 in the inclusion bodies of differentiating Skp1-deficient cells. Scrambled and lentiviral-stable infected *SN4741* cells were induced to differentiate as in Fig. 13. Cells were fixed and double-stained with anti-Hsc-70 (red) and anti-ubiquitin (green) antibodies. **A** Scrambled-infected cells (**B**) *SKP1A*-deficient cells developed inclusion bodies (arrows) 24 h after induction of differentiation. Regions of co-localization within cells stain yellow (right-hand panel). Each panel shows a representative picture of 10–15 views in two independent experiments

Excessive accumulation of misfolded proteins and inhibition of proteasomal function are considered to promote formation of aggresome/LB-like inclusions and toxicity in cultured DAergic neurons (Cookson et al. 2003; Wang et al. 2005). *SN4741* neurons infected with* SKP1A* shRNA-1 and grown at permissive temperature did not show any cytoplasmic aggregates, similar to cells infected with the scrambled vector (Fig. 13A, C). However, *SKP1A*-deficient cells under the deleterious differentiation process develop cytosolic round inclusion structures which stain positively for α-synuclein, TH, ubiquitin, proteasomal ATPase subunit PSMC4, and Hsc-70 [Fig. 13B, D, E (arrows) and Fig. 14], all of which are components of LBs (Marx et al. 2007; Auluck et al. 2002; Jellinger 2003). Some of them appeared as a single perinuclear inclusion aggregate (insets), reminiscent of centriole-associated structures termed aggresomes (McNaught et al. 2002; Wileman 2007), characterized by the presence of intermediate filaments such as γ-tubulin, (Fig. 13F), which co-localized with ubiquitin and the proteasome subunit, PSMC4 (Fig. 13F, G). Quantitative analysis indicated that 42.7% ± 4.3 and 46.8% ± 5.1 of Skp1-deficient cells bear inclusion bodies co-staining for α-synuclein/TH and α-synuclein/ubiquitin, respectively. Summarizing the recent findings we have provided supporting evidence that the deficiency in *SKP1A* induced a lethal phenotype only in arrested/differentiated SN-derived DAergic cells exhibiting proteinaceous round inclusion structures, which were almost identical in composition to human LB. To the best of our knowledge, this is the first evidence that a decrease in the endogenous levels of an E3 ubiquitin ligase, identified as significantly reduced in human SNpc from SPD, promotes the formation of LB-like structures and ultimate cell death, occurring only in differentiated neurons.

### Gene–gene/gene–environment interactions

#### Single/double knockdown of *SKP1A* and *ALDH1A1* genes and increased susceptibility to exogenous insults

The present findings have shown that major features of DA neuron pathology were replicated by *SKP1A* gene deficiency in *SN4741* DAergic cell line. These include morphological and cell cycle abnormalities, down-regulation of DAergic markers and an inability of the cells to arrest when induced to differentiate, expressing round aggregates reminiscent of LBs and culminating in cell death. According to the ‘dual-hit’ hypothesis of neurodegenerative diseases (see Research hypothesis), it is predicted that gene–gene and/or gene–environmental factors would act in concert or sequentially to propagate the pathological process of PD. To challenge this hypothesis, we have: (a) exposed Skp1-silenced cells to environmental stressors that have been incriminated in PD DAergic neurodegeneration; (b) selected to knock down *ALDH1A1* on top of the *SKP1A*-defficient *SN4741* cells to determine its impact as a modifier of *SKP1A*-mediated phenotype in response to the same external stressors.

Aldh1 is a central player in DAergic function owed to its participation in the degradation of aldehyde derivatives of DA, generated by the action of MAO-A and -B (Mardh and Vallee 1986). These highly reactive, neurotoxic aldehydes can accumulate in case of decreased levels of Aldh1, as occurring in SNpc of PD (Galter et al. 2003; Mandel et al. 2007), and can promote neuronal death (see the section “Gene expression profiling of SPD SN”). In our human PD SN transcriptomic and proteomic studies, we have found that both Skp1 and Aldh1 gene and protein levels were dramatically reduced (Grunblatt et al. 2004). Further support for Aldh1 involvement in DAergic neuron maintenance is provided by our in vitro findings (Fig. 4), where the protein levels of Aldh1, together with those of Skp1 and Hsc70 were early and progressively down-regulated in naïve *SN4741* cells upon exposure to MPP^+^.

**Fig. 15:**
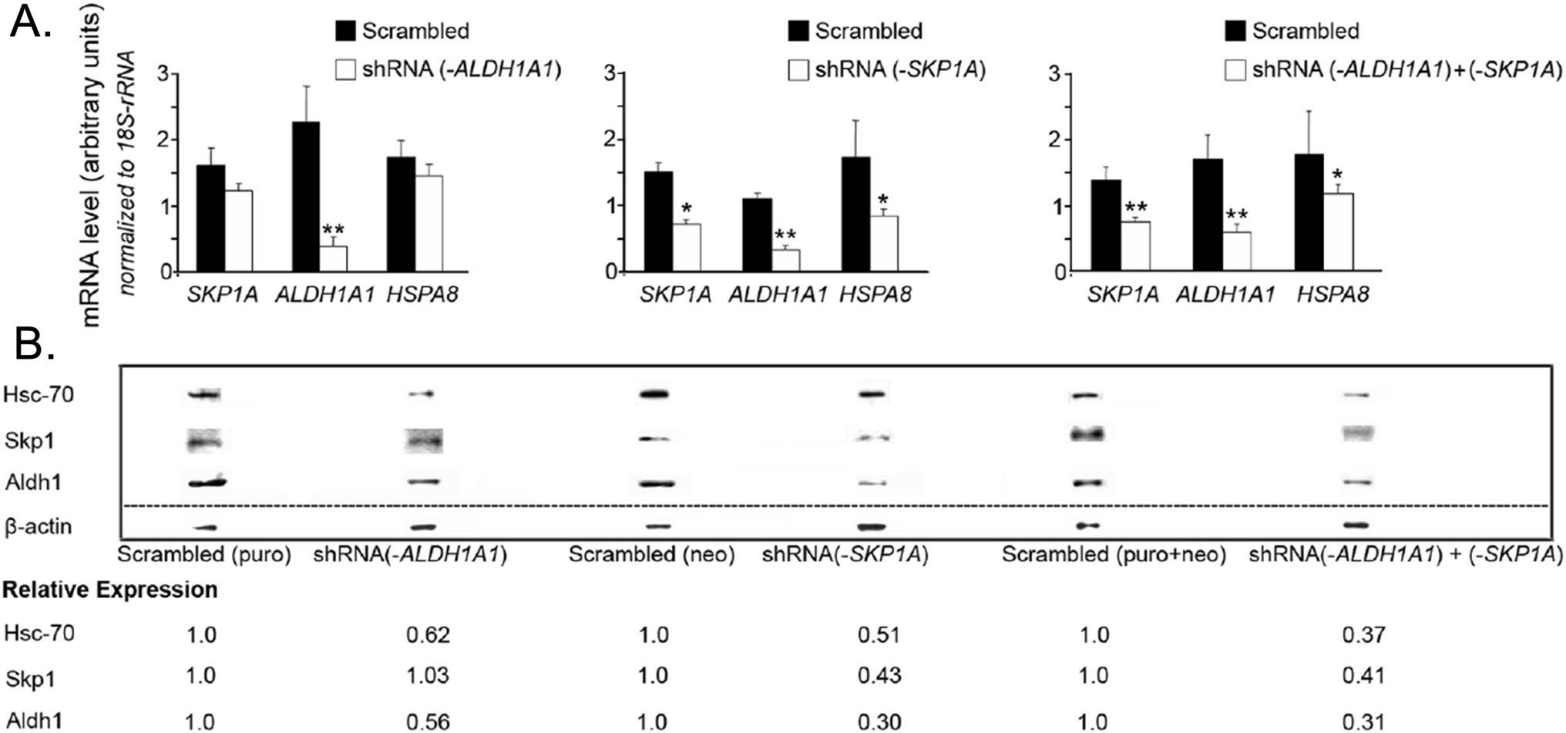
RNAi-mediated silencing of *SKP1A* and/or *ALDH1A1* in *SN4741* cell line. **A** *SN4741* cells were infected for 1 week with LVs’ plasmid vectors encoding shRNAs targeting *SKP1A*, *ALDH1A1* or a Scrambled (neo), Scrambled (puro) sequences, respectively. The shRNA(*-SKP1A*) transfected clone was subsequently infected with a second vector carrying the *ALDH1A1* gene sequence, with a final permutation of two separate genes: shRNA(*-ALDH1A1*) + shRNA(*-SKP1A*). RNA was extracted and converted to single stranded for analyzing by Q-PCR. Following infections, the mRNA levels of *SKP1A*, *ALDH1A1*, *HSPA8* (encoding Hsc-70), and *VMAT2* were quantified by Q-PCR. The relative expression level was assessed by normalizing to the housekeeping genes *18S-rRNA*. Values are mean ± SEM from three independent experiments conducted in two replicates. *p < 0.05; **p < 0.01 *vs*. scrambled vectors. **B** After lentiviral infection cells, homogenates were analyzed by Western blotting using Skp1, Aldh1, and HSC-70 specific antibodies. Bands were quantified by densitometry and normalized to β-actin

To obtain *SKP1A-ALDH1A1* double knockdown cells, an shRNA(*-SKP1A*) transfected clone was subsequently infected with a second vector carrying shRNA (*-ALDH1A1*) gene sequence under the selection of both puromycin and neomycin. In parallel, individual silencing of *ALDH1A1* and *SKP1A* genes by RNAi was performed as well as transfection with their respective scrambled vectors, serving as controls. Q-PCR analysis revealed a significant decrease in mRNA of *ALDH1A1* (~ 83% reduction) caused by shRNA(*-ALDH1A1*) infection compared to scrambled shRNA-infected cells (Fig. 15A, left panel). Western blot analysis showed also a reduction (44%) in the expression levels of Aldh1 protein (Fig. 15B). In addition to the observed down-regulation in *SKP1A* mRNA transcript and protein levels induced by shRNA-mediated silencing of *SKP1A* (48% and 57% reduction, respectively), the single deficiency of *SKP1A* caused a robust reduction in the expression levels of *ALDH1A1* gene and protein (~ 70% of control, Fig. 15A and B, middle panel), providing evidence for an intimate connection between Skp1 and Aldh1.

Conversely, no significant alteration in *SKP1A* mRNA transcript or protein levels was seen when *ALDH1A1* was silenced by RNAi (Fig. 15A and B, left panel). These results suggest a common biological pathway shared by both genes, posing Skp1 upstream of Aldh1 in the hierarchy of DAergic function. As expected, the double gene transfection led to a reduction in their transcripts and protein levels similar to that exerted by the individual gene knock downs (Fig. 15A and B, right panel).

##### Individual or simultaneous SKP1A-ALDH1A1 knockdown increases SN4741 neuron sensitivity to serum deprivation

We evaluated the vulnerability of Skp1-, Aldh1-, and Skp1/Aldh1-deficient cells to serum withdrawal, an established model of slowly progressive neuronal death (Batistatou and Greene 1993; Macleod et al. 2001). Local neurotrophic support is indispensable for the maintenance of neuronal cells and neurons deprived of serum or trophic factors undergo death via apoptosis (Batistatou and Greene 1993; Troy et al. 2001). Serum starvation led to a time-dependent loss in cell viability of scrambled vector (neo)-infected control cells, of 71.0 ± 0.5% and 55.9 ± 0.2% *vs.* control (2% FCS) after 48 and 72 h, respectively.

**Fig. 16:**
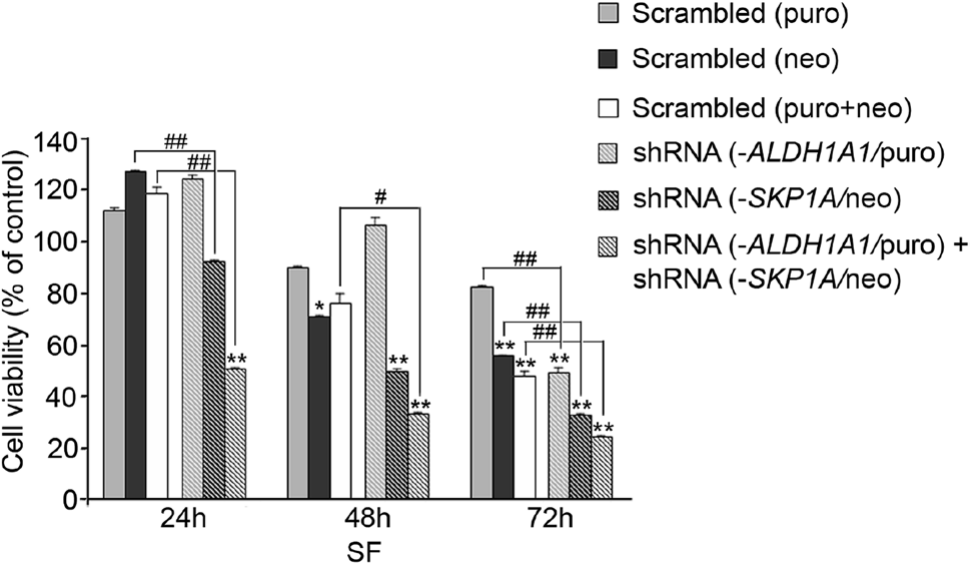
Effect of trophic factors support withdrawal on *SKP1A* and/or *ALDH1A1*-silenced *SN4741* cells. **A** Lentiviral cells were seeded in 2% FCS containing medium for 24 h, and subsequently deprived of serum support for a period of 24–72 h. Cell viability was assessed by the MTT test. The results are the mean ± SEM of a representative experiment conducted in seven replicates. (*) vs. respective control (2% FCS); (#) *vs.* scrambled. 1 sign (*#): *P* < 0.05, 2 signs: *p* < 0.01

The deficiency of Skp1 promoted a further susceptibility to serum starvation reducing cell viability to (50.0 ± 0.9% and 33.0 ± 02%; *vs*. control (2% FCS) after, 48 and 72 h, respectively (Fig. 16). However, the most salient effect was obtained after double knocking down of *SKP1A* and *ALDH1A1* which provoked a synergistic reduction in cell survival already visible at 24 h post-serum starvation (50.7 ± 0.4% of 2% FCS control) and persisted along the 3d period (33.6 ± 0.4% and 24.6 ± 1.2% after 48 and 72 h, respectively; Fig. 16). On the contrary, the viability of *SN4741* cells deficient in *ALDH1A1* was not compromised until a 72 h period of serum starvation (49.4 ± 1.7% of c, Fig. 16). This gene–gene manipulation supports the possibility of an existing cross-talk between Skp1 and Aldh1 that is essential for DAergic function.

##### Effect of mitochondrial complex I inhibition on survival of individual or double SKP1A-ALDH1A1 knocked down DAergic cells

**Fig. 17:**
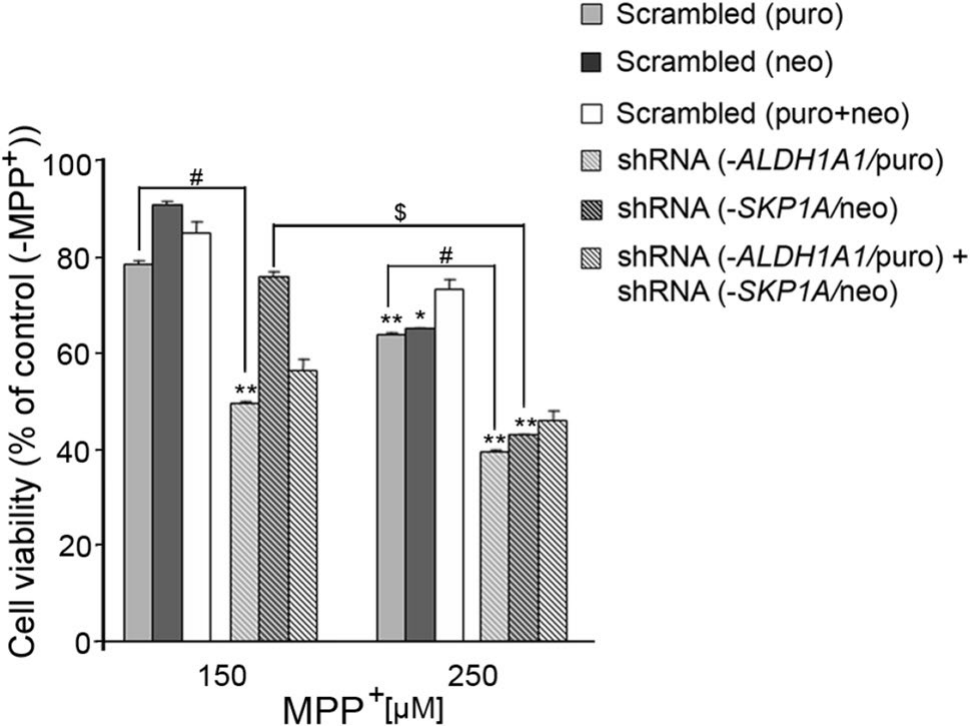
Effect of MPP^+^ on *SKP1A* and/or*ALDH1A1*-silenced *SN4741* cells. *SKP1A* and/or *ALDH1A1*-silenced and non-silenced cells were seeded in 10% FCS containing medium for 24 h, and then, fresh medium with 0.5% FCS and 150 or 250 μM of MPP^+^ was added for an additional 48 h. Cell viability was assessed by the MTT test. The results are the mean ± SEM of a representative experiment conducted in seven replicates. (*) *vs*. respective control (-MPP^+^); (#) *vs*. scrambled at same MPP^+^ concentration; ($) *vs*. shRNA at 150 μM MPP.^+^.1 sign (*#$): *P* < 0.05, 2 signs: *p* < 0.01

**Fig. 18:**
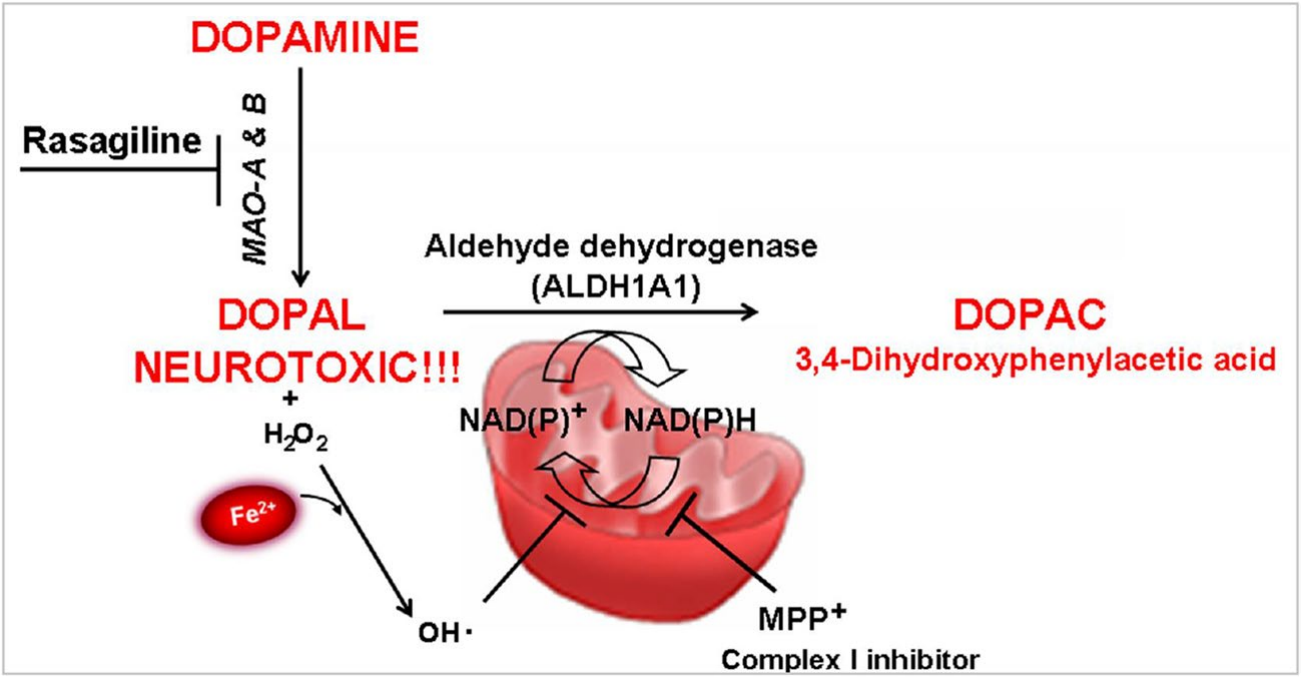
Detoxification by *ALDH1* following high DA concentrations and prevention by Rasagiline. DA has been implicated as an endogenous neurotoxin to explain the selective neurodegeneration as observed for PD. DA undergoes catabolism by MAO to 3,4-dihydroxyphenylacetaldehyde (DOPAL), which is further oxidized to 3,4-dihydroxyphenylacetic acid (DOPAC) via aldehyde dehydrogenase (*ALDH*). *ALDHs* are a group of nicotinamide adenine dinucleotide (NAD(P)^+^)-dependent enzymes that catalyze the oxidation of aldehydes to their corresponding carboxylic acids (Marchitti et al. 2007). The catecholamine-derived aldehyde, DOPAL, is a neurotoxin and its intraneuronal accumulation is involved in cell death associated with neurodegenerative, including PD and AD (Burke 2003; Mattammal et al. 1995), and can be detoxified by various enzyme systems including *ALDH*, which is exclusively responsible for their oxidative metabolism. MPP^+^, a complex I inhibitor may impair the activity of *ALDH* by interfering with the reduction of its cofactor NAD(P)^+^ to NAD(P)H by the electron transport flux. A mitochondrial complex I deficit has been identified in the SN of PD patients (Schapira et al. 1990) and hypothesized to result from genetic mutations and/or environmental toxins (Bachurin et al. 2003; Fiskum et al. 2003). Furthermore, Rasagiline (N-propargyl-1R-aminoindan) highly potent irreversible MAO-B inhibitor anti-PD drug

In the previous section, we examined the response of the single- or double-silenced cells to damage induced by depriving cells of serum support. This is considered to cause broad-spectrum cell damage, since the crucial trophic factors, nutrients, and metals present in serum are important activators/players in an array of survival pathways and cell metabolism. We next concentrated on the selective impairment of mitochondrial function, as mitochondrial dysfunctions, including complex I deficit, are associated with neurodegenerative diseases (Robinson et al. 1998; Tatton and Olanow 1999) and have been identified in the SN of PD patients (Schapira et al. 1990). For this purpose, the different cell clones were exposed to MPP^+^ neurotoxin, an inhibitor of complex I of the mitochondrial electron transport chain, which leads to the buildup of free radicals and toxic molecules that contribute to cell destruction. MPP^+^, at a concentration that did not elicit a visible damage to the control cells (150 µM), significantly decreased the viability of the *ALDH1A-* and *ALDH1A1/SKP1A-*silenced cells (49.6 ± 0.3% and 56.4 ± 2.3% of control (-MPP^+^), respectively; Fig. 17), while no such effect was seen in the *SKP1A-*knocked down cells. At a higher concentration (250 µM), the extent of the toxicity was basically similar for the three cell clones. The selectivity of the insult to *ALDH1A1-*deficient cells may result from a synergistic action of a shortage in the availability of the cofactor of Aldh1, nicotinamide adenine dinucleotide (phosphate) [NAD(P)^+^], resulting from an impairment in the mitochondrial electron transport flux caused by MPP^+^ (Fig. 18), and from a deficiency of the enzyme.

##### DA exposure increases sensitivity of SKP1A or ALDH1A1-silenced cells

**Fig. 19:**
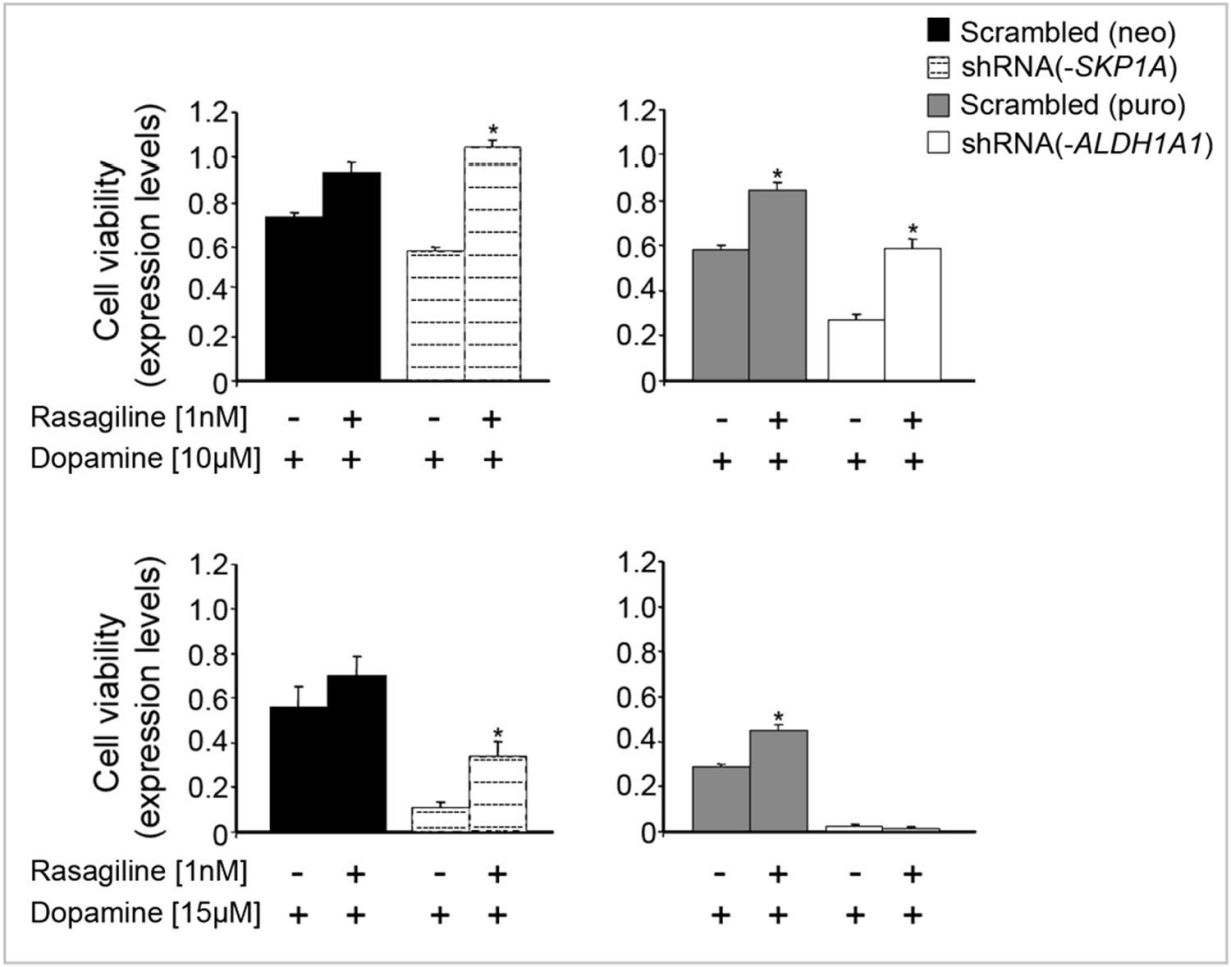
Effect of Rasagilin on DA-induced toxicity in* SKP1A* and/or* ALDH1A1*-silenced cells. *SKP1A* and/or *ALDH1A1*-silenced and non-silenced cells were seeded in DMEM medium-containing 10% FCS for 24 h. Then, Rasagiline (1 nM) was added 30 min before exposure to DA (10 or 15 μM) under 2% FCS for an additional 24 h. Cell viability was assessed by the MTT test. The results are the mean ± SEM of a representative experiment conducted in seven replicates. *p < 0.01 *vs*. respective control (Rasagiline drug (Youdim et al. 2001) can block the metabolism of DA, therefore reducing the accumulation of DOPAL

DA has been implicated as an endogenous neurotoxin to explain the selective neurodegeneration as observed in PD. DA undergoes catabolism by MAO to 3,4-dihydroxyphenylacetaldehyde (DOPAL), which is further oxidized to 3,4-dihydroxyphenylacetic acid (DOPAC) by Aldhs. Aldhs are a group of NAD(P)^+^-dependent enzymes that catalyze the oxidation of aldehydes to their corresponding carboxylic acids (Marchitti et al. 2007). The catecholamine-derived aldehyde, DOPAL, is a neurotoxin and its intraneuronal accumulation is involved in cell death associated with neurodegenerative, including PD and AD (Burke 2003; Mattammal et al. 1995). It can be detoxified by various enzyme systems including Aldh, which is exclusively responsible for their oxidative metabolism (Fig. 18). As shown in Figs 10 and 19, 15 µM of DA decreased cell viability of control, scrambled (neomycin or puromycin) vectors-infected cells after 24 h [73.8 ± 1.6% and 56.2 ± 8.9% or 58.2 ± 1.9% and 29.1 ± 1.2% of control (-DA), respectively]. A more pronounced decline in cell viability was observed in *ALDH1A1*-silenced cells exposed to both concentrations of DA (26.6 ± 3.1% and 2.8 ± 1.2% of control (-DA) for 10 and 15 µM of DA, respectively (Fig. 19, right panel). Skp1 knocked down cells also displayed an increased vulnerability to DA exposure compared to the scrambled control, probably resulting from its negative effect on *ALDH1A1* expression [57.9 ± 2.3% and 10.8 ± 2.9% of control (-DA) for 10 and 15 µM of DA, respectively; Fig. 19, left panel]. This stands in agreement with a role of Aldh1 in the detoxification of DA metabolites.

The potential toxicity of DA can be reduced either through the oxidation of its metabolite DOPAL to the corresponding carboxylic acid (DOPAC), catalyzed by Aldh, or abrogated by the pharmacological action of a MAO inhibitory agent. Indeed, pretreatment for 30 min with the anti-Parkinson drug Rasagiline (0.1 nM), an MAO-B inhibitor with neuroprotective properties in PD (Mandel et al. 2005a, b; Weinreb et al. 2010), significantly increased the cell survival index (twofold over non-treated cells) of *ALDH1A1*-deficient cells at 10 µM of DA, but rasagiline could not overcome the massive cell death (98%) induced by 15 µM of DA (Fig. 19, right panel). Similarly, rasagiline was effective against DA exposure of scrambled infected cells, with respective improvements in cell viability of 1.4- and 1.5-fold for each concentration (10 and 15 µM, Fig. 19). Also, the inhibitor almost duplicated or tripled the survival index of *SKP1A* silenced cells at 10 and 15 µM of DA, respectively (from 57.9 ± 2.3% to 104.8 ± 2.8% and from 10.8 ± 2.9% to 33.9 ± 6.5%, respective to each concentration; Fig. 19, left panel).

### Development of a novel mouse model of SPD

The identification of Skp1 as a key player in DA neuron function suggested that a targeted site-specific reduction of Skp1 levels in mice SNpc, may result in a progressive loss of DAergic neurons and terminal projections in the striatum. To this aim, site-specific RNAi with lentiviral (LV) shRNA (LVshRNAi) carrying vector was performed by injecting 10 mice bilaterally into the mouse SNpc with LV-*SKP1A* (*n* = 5) or LV-scrambled (control group, *n* = 5).

#### Behavioral assessments of motor disabilities in mice injected with *SKP1A* LVshRNAi carrying vector targeted to the SNpc

**Fig. 20:**
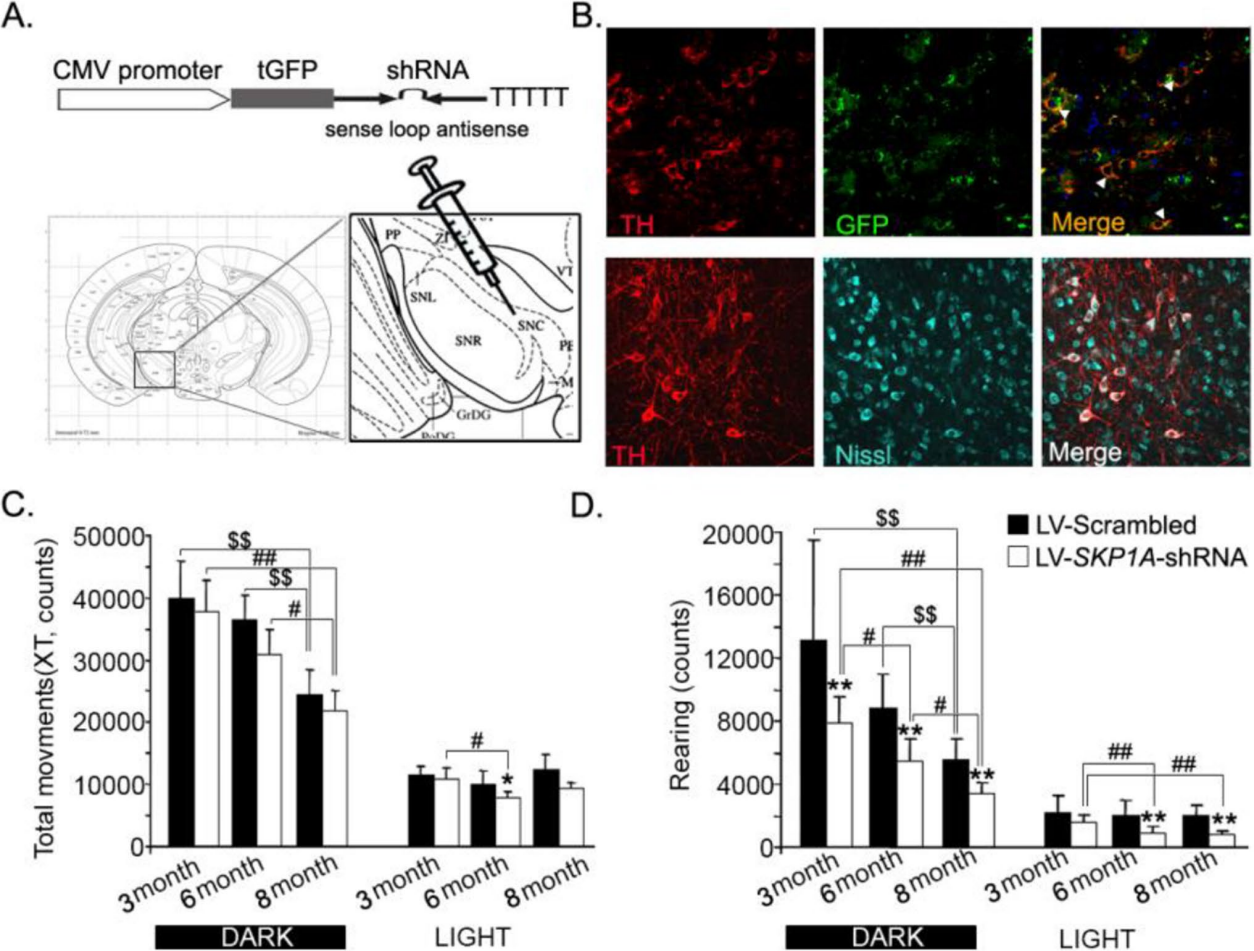
Stereotactic injection of LV-*SKP1A*shRNA into adult SNpc and locomotor deficits. **A** Schematic representation of plasmid constructs and site-specific LV delivery. **B** Upper panel: antibodies against the LV reporter GFP and TH confirmed a specific LV localization to the SN in a control mouse. Lower panel: TH-positive neurons co-express Nissl dye and display normal morphology inside the SN indicating LV infection is not deleterious to the neurons. **C** General locomotion (XT, horizontal movements) was measured at 3–8 month post-LV injection, for 2 consecutive days in home cage. The graph presents two 12 h dark–light cycles. No differences were found in the total activity between the groups both in the light and the dark phase of the day. **D** Age-dependent decline in rearing was evident in LV-*SKP1A* mice compared to control in both dark–light cycles. 1 sign (*$#): *P* < 0.05, 2 signs: *p* < 0.01. (*): *vs*. control (LV-scrambled) at same time-point

Mice were followed for the temporal progression of motor disability up to 8 months post-injection. Then, mice were perfused intracardially and their brains were sectioned. Using a first antibody against the LV reporter GFP and streptavidin Cy2 conjugated as secondary antibody, we confirmed a specific LV localization to the TH-positive neurons in the SNpc in all but one mouse in both groups, which were omitted from the analysis (Fig. 20B, control. Upper, orange in overlaid panel: white arrowheads). Furthermore, Nissl staining for living cells revealed that all TH-positive neurons inside the SNpc are viable (Fig. 20B, control. Lower, white neurons in overlaid panel) and exhibit normal gross morphology and arborization, indicating that LV infection is not deleterious to the neurons. LV-*SKP1A* injected mice showed no significant difference in general locomotion (XT, horizontal movements) compared to control (LV-scrambled), but both groups experimented a comparable age-dependent decline up to ~ 40% at 8 months post-injection (Fig. 20C). Although the rearing behavior (vertical movement) also declined with age, Skp1-deficient mice reared less from the first measurement (3 months) exhibiting a significant difference in the number of rearing events compared to control mice both in the light and the dark phase of the day, which accentuated progressively with time. At 8 months, the numbers of rearing events in the Skp1 deficient mice were reduced to 43% of those seen at 3 months (Fig. 20D). No significant changes in mouse weight were observed. The reduction of the vertical movements, that is rearing, may be reminiscent of the early occurrence of hypokinesia and axial, postural instability in PD.

#### Integrity of the nigro-striatal axis by tracking neurochemical deficits and histopathology of the SN and striatum

**Fig. 21:**
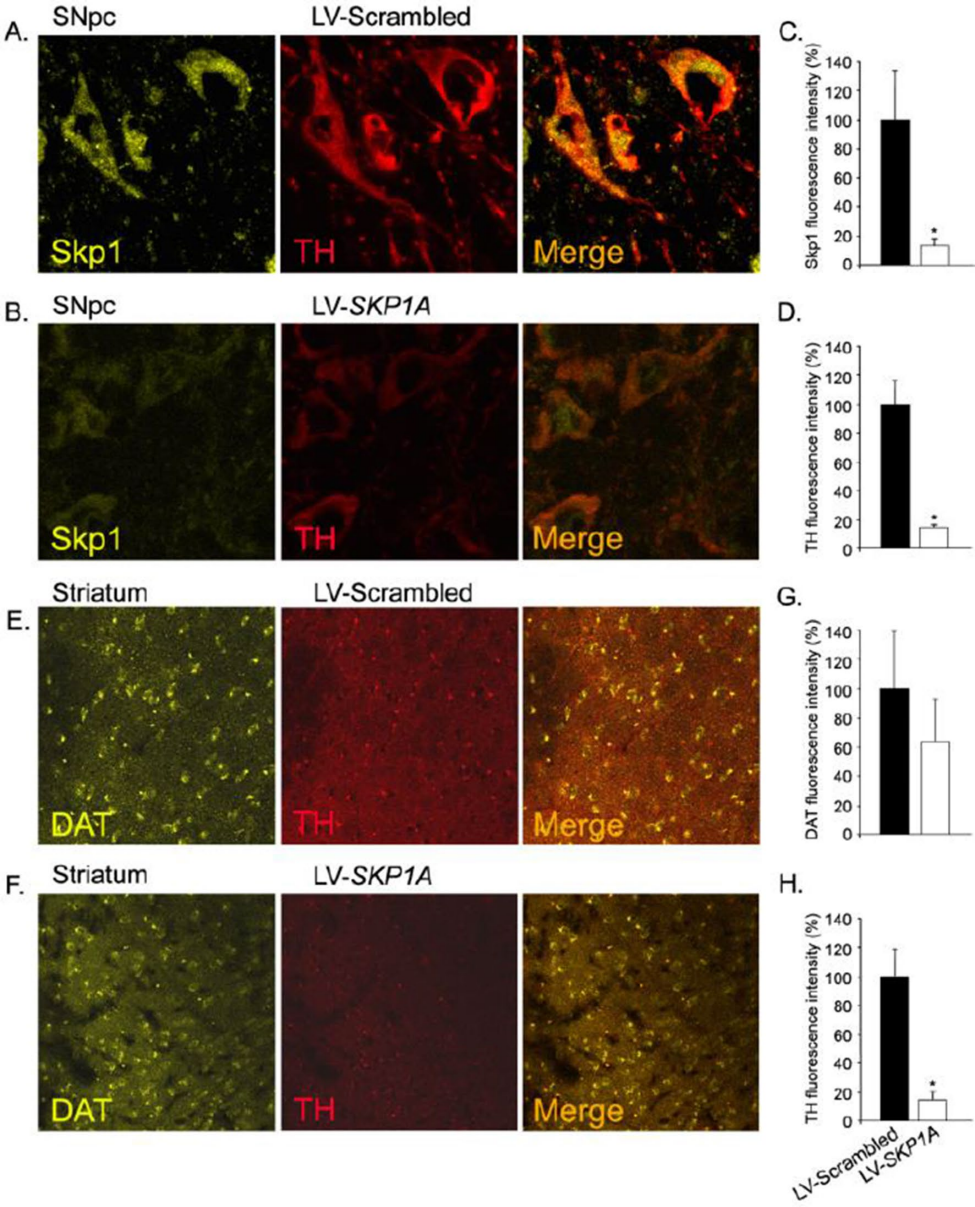
Expression of Skp1 and DAergic-specific markers (TH and DAT) in the SNpc and striatum of mice injected with *SKP1A* or scrambled shRNA lentiviral vectors. **A**,**B*** LV*-*SKP1*A infection led to significant reduced expression of Skp1 protein levels with a parallel loss of TH-IR, 8 months post-injection (× 63). **C**,**D** Histograms representing the average loss of Skp1 and TH fluorescence intensity in all mice. **E**,**F** *LV*-*SKP1*A infection led to significant reduced expression of DAT-IR in striatum and a more drastic decline of TH-IR (× 25). (**G**,**H**) Quantification of the striatal DAT and TH labeling intensity in the two groups. *P < 0.05, nonparametric Kruskal–Wallis ANOVA followed by the Mann–Whitney two-tailed *U* test

Following the time-course of locomotion deficits (8 months post-viral injection), mice were perfused intracardially and their brains were sectioned. Representative images depicting double labeling with TH and Skp1 antibodies and optical density quantification from all mice selected sections show that in control brains, TH-immunoreactive (IR) nigral neurons show a robust expression of Skp1 (Fig. 21A, C, yellow–orange). On the other hand, LV-*SKP1A* infection led to significant reduced expression of Skp1 protein levels of more than 85%, indicating that the site-directed LV transduction was successful (Fig. 21B, C). Remarkably, this deficiency caused a parallel drastic loss (85.4%, Fig. 21D) of TH-IR, that was accompanied by a comparable decline of TH expression in the striatum (Fig. 21E, F, H). There are some indications that the damage to the neurons resulting from Skp1 deficiency may have been initiated at early stage: staining of striatal tissue with antibody against the DA transporter, DAT which is indicative of the integrity of DAergic terminals reveals a 36% decrease in DAT-IR (Fig. 21E–G); also, the gradual age-dependent decline in the rearing behavior (vertical movement) suggests an early impairment in some motor aspects.

In summary, a targeted site-specific reduction of Skp1 protein levels in mice SNpc have resulted in a progressive loss of DAergic neurons and terminal projections in the striatum, and recreated motor disabilities.

## Discussion

The main purpose of this review was to shed light on the relevance of Skp1 to PD pathogenesis. The results have identified Skp1 as a fundamental player in DA neuron function, differentiation, and survival. Indeed, its deficiency was lethal to arrested/post-mitotic SN-derived cell, promoting similar molecular alterations as those described in SPD. A major important finding was that *SKP1A*-deficient mice clearly demonstrated time-dependent motor disabilities and robust loss of TH-immunoreactivity in the SNpc and striatum in adult mice.

Our experiments provide evidence that the genetic vulnerability caused by knockdown of *SKP1A* renders DA neurons especially sensitive to genetic reduction of Aldh1 and external stressors, which have been implicated in PD pathology.

If successful, the availability of a model of SPD that can replicate the adult-onset, progressive neurodegeneration in specific tissues and motor deficits will provide a reliable "platform" to develop new therapeutic interventions.

The following sections discuss point by point the main findings of the study and their implications regarding the potential for understanding disease pathology and consequent therapeutic treatment.

### Regulation of Skp1 in vitro and in vivo models of PD

Our large-scale transcriptomics and confirmatory proteomic analyses in human post-mortem tissue have shown that* SKP1A* gene and protein are significantly reduced in the SNpc of PD patients, compared to age- and gender-matched controls without neurological disorders (Grunblatt et al. 2004). This was accompanied by parallel reductions in *DA*T, *VMAT2*, *HSPA8*, and *ALDH1A1*, playing central roles in the processing of aberrant proteins, regulation of DA disposition in the synapse and cytosol, and degradation of toxic aldehyde derivatives of DA. The human impairments were, in part, replicated in the MPTP model of PD where a significant decrease of Skp1 was particularly observed in the midbrain in correlation with a loss of TH protein. Furthermore, our results demonstrated that the midbrain and striatum contain the lowest Skp1 protein levels, compared to frontal cortex, hippocampus, and cerebellum, probably contributing to the particular susceptibility of the SNpc DAergic neurons to different types of stresses such as increased iron concentration, enzymatic (monoamine oxidase) and non-enzymatic (auto-oxidation) DA metabolism, abnormal protein accumulation, proteasomal inhibition, and neurotoxin-induced OS.

In agreement with our findings in humans and MPTP-induced parkinsonism, cultured naïve *SN4741* DA neurons exposed to the DA neuron selective neurotoxin MPP^+^, a dynamic producer of ROS, caused an early and progressive down-regulation of Skp1, concomitantly with Aldh1 and Hsc-70 proteins. These finding supports the view of these proteins as intimately connected to the neuronal death process in SPD and probably, acting as early players in the neurotoxic cascade. It has been proposed that subtle alterations in a small number of interconnecting crucial genes, occurring during the pre-symptomatic manifestation of PD, account for the slowly progressive neurodegeneration of the DA-containing neurons (Mandel et al. 2007). It is possible that the gradual functional decrease in Skp1 and its DAergic counterparts shown in this study represents a “convergence point” shared by both the familial and sporadic cases.

Further supporting a defensive role, overexpression of Skp1 protein conferred protection to naïve *SN4741* DAergic cells against damage induced by exposure to MPP^+^ and pharmacological inhibition of the proteasome. The observation that Skp1 is still able to rescue *SN4741* cells under proteasomal inhibition implies a distinct function of Skp1 than as an E3 ubiquitin ligase. In this regard, it has been lately shown that Fbx2 (Fbs1), a brain-enriched F-box protein appears to function with Skp1 in a novel heterodimeric complex (without cullin 1), rather than in the traditional SCF tetramer in inner ear cochlear mammalian tissue and in isolated cell culture (Nelson et al. 2007; Yoshida et al. 2007). It has been suggested that Fbs1 assists clearance of aberrant glycoproteins in neuronal cells by suppressing aggregates formation, thus functioning as a unique chaperone for those proteins in addition to the role of the SCF^Fbs1^ ubiquitin ligase, opening new perspectives for cellular activities of F-box proteins (Yoshida et al. 2007).

### The impact of a deficiency in *SKP1A* on DAergic phenotype: cell culture study

The possible neuroprotective role of Skp in DA neurons was further illustrated in cell deficient in Skp1. The shRNA-mediated decrease of Skp1 in *SN4741* neurons induced parallel down-regulations of the DAergic neuron phenotype markers *DAT*, *VMAT2*, and *ALDH1A1* mRNAs and a slight but significant decrease in *HSPA8* transcript. Given that *ALDH1*, *VMAT* and *DAT* are located within DA-containing neurons of SNpc, the reduction in their gene expression may contribute to a failure in DA transmission and metabolism. The correlation between the identified gene changes depicted in cells deficient in Skp1 or damaged with MPP^+^ and those in human PD SNpc DA neurons further supports the assumption that Skp1 may play a key role in DAergic neuron differentiation. More evidence is provided by the prominent elevation in both *SKP1* gene and protein levels upon cell cycle arrest-induced differentiation of naïve neurons, in parallel to the DA neuron markers *DAT*, *TH*, *VMAT2*, and *ALDH1A1*. As far as we know, this is the first report of Skp1 as a potential player in neuronal maturation. However, the most striking observation stressing the potential role of Skp1 as a determinant of DAergic neuron phenotype was the finding that its deficiency was lethal only to arrested/post-mitotic SN-derived cells. Collectively, our results links, for the first time, Skp1-to-DA neuronal function and survival suggesting an essential role in SPD.

There are at least two possible explanations for the lethal phenotype demonstrated in *SKP1A* silenced, arrested cells: the reduced expression of Skp1 protein may cause the malfunction of the E3 ligase SCF complex and subsequent dysregulation of cell cycle regulators of the G_1_/S transition, such as cyclins, CDKs, and cell cycle inhibitors (Feldman et al. 1997; Spruck and Strohmaier 2002). It is well known that in neurodegenerative diseases, there is a loss of cell cycle control, leading eventually to neuronal death (Copani et al. 2001). Indeed, events that force a mature neurons back in to the cell cycle are lethal rather than mitogenic for the neuron. In line with this, knockdown of two components of the UPS, the ubiquitin ligase scaffolding protein Cul-1 and the proteasome-associated deubiquitinating protein Pad-1, leads to cell cycle reactivation and apoptosis in subsets of post-mitotic neurons (Staropoli and Abeliovich 2005).

Alternatively, but not mutually exclusive, Skp1 might have a structural or functional role in the cell, essential to cell survival and differentiation processes in higher organisms. A first functional link between Skp1 and neurodegeneration in mammals has been provided only lately in mice null for Fbx2, a brain-enriched F-box protein that appears to function with Skp1 in an apparently novel heterodimeric complex rather than in the traditional SCF tetramer in inner ear cochlear tissue. Here, the deficiency of Fbx2 led to a parallel loss of Skp1 specifically in the cochleae and cellular degeneration resulting in age-related hearing loss (Nelson et al. 2007). There was no indication however, regarding the expression levels of Skp1 in the SN of these Fbx2 null mice and whether the integrity/function of the DA neurons was compromised. Consistent with this finding is that in yeasts, Skp1 was found to associate with the F-box protein RCY1 without assembling into an SCF complex, and to participate in the recycling of internalized proteins (Galan et al. 2001). Parkin has been found to function in an SCF-like multiprotein complex that includes the F-box/WD repeat protein hSel-10 and Cullin-1 and cyclin E was identified as a target of this complex (Staropoli and Abeliovich 2005). This composite activity of parkin may potentially increase the repertoire of proteins targeted for ubiquitination. A second link between Skp1 and neuronal function was lately provided by the identification of parkinsonism-causing mutations in *PARK15*/*FBXO7* (Di Fonzo et al. 2009), a Skp1-interacting protein. Fbxo7 physically interacts with Skp1 for assembling into an SCF-E3 ubiquitin ligase, and is involved in crucial processes such as apoptosis (Chang et al. 2006).

The fact that humans express only one functional Skp1 isoform (Semple 2003) combined with the decreased expression in PD SNpc may account for the wide impairment in the function of proteins implicated in DAergic neurotransmission and in the accumulation of a wide spectrum of ubiquitinated protein aggregates in brains of PD patients, such as TH, synphilin-1, α-synuclein, and phosphorylated tau. Indeed, in addition to the massive lethality of arrested/differentiated SN-derived DAergic cells, we found at the cellular level proteinaceous round inclusion structures which stained positively for α-synuclein, TH, ubiquitin, 19S proteasomal protein PSMC4 and Hsc-70, all components of LBs. Some inclusions presented as single perinuclear γ-tubulin-positive aggregates, reminiscent of centriole-associated structures termed aggresomes (Olzmann et al. 2008). Likewise, directed overexpression of α-synuclein in Drosophila induced formation of perinuclear inclusions similar to LBs that stained positive to α-synuclein and the chaperone Hsp70, implicating the molecular chaperone machinery in the pathogenesis of PD (Auluck et al. 2002). It is tempting to speculate that a similar process may occur in the DAergic neurons of PD patients, where these structures may represent a precursor of LBs. As far as we know, this is the first report showing that a deficiency in an E3 ubiquitin ligase, identified as significantly reduced in human SNpc from SPD, promotes the formation of aggresome-like inclusions and ultimate cell death, occurring only in differentiated neurons. Thus, the present finding can suggest a novel paradigm in which dysfunction of Skp1 promotes LB-like inclusion body formation, rather than the presence of these inclusions as the culprit of DA neuron death. Interestingly, in parkin-related PD, there is a general absence of LB inclusions in post-mortem brain samples of patients, and SPD brains do not present alterations in the mRNA or protein levels of Parkin.

Although Skp1-deficient neurons begin to die in the process of differentiation, morphological and biochemical changes were already apparent in proliferating cells. The cells acquired an elongated and thinner cell body and divide more slowly because of a delay in completion of the cell cycle.

Summarizing the in vitro studies, the results suggest that Skp1 plays a fundamental role in DA neuron viability in the context of cell cycle arrest and differentiation. Indeed, its deficiency has reproduced to a significant extent, the molecular alterations described in SPD at the cellular level. This and the protection afforded by Skp1 overexpression support its genetic manipulation as a new model of SPD. The understanding of the functional/structural role of Skp1 and its mechanism of action may lead to novel therapeutic approaches targeted at its molecular pathway counterparts.

### Gene–gene/gene–environment interactions

SN neurons live in an oxidative stress environment (Jenner 2003), with high concentrations of iron, neuromelanin, and DA (Barzilai and Melamed 2003), and may have life-long exposure to factors (environmental and intrinsic, e.g., toxins, genetic susceptibility, and aging), that may increase the risk of PD. This is believed to invoke a gradual occurrence of subtle alterations in one or more parameters of cell vitality/function that may initiate and propagate disease pathology. Thus, we predicted that the genetic deficiency in *SKP1A* will render the DAergic cells more vulnerable to damage induced by intrinsic, or extrinsic factors or both. Indeed, our findings are compatible with the ‘dual-hit’ hypothesis of neurodegenerative diseases, as exposure of the *SKP1A-*knocked down cells to external insults implicated in SN DAergic neuropathology, such as decreased support of neurotrophins or MPP^+^ (gene–environment interaction), exacerbated the induced damage compared to scrambled vector infected cells. In the same context, knockdown of *ALDH1A1* gene on top of the *SKP1A*-deficient *SN4741* cells (gene–gene interaction) increased their sensitivity to the above external insults, compared to the individual silenced cell clones, indicating that *ALDH1A1* is a modifier of *SKP1A*-mediated phenotype and probably shares a common biological pathway. This assumption receives support from the observation that *SKP1A* RNAi brought up a decline in the levels of the *ALDH1A1* mRNA transcript. This action was, however, not reciprocal as the individual knockdown of *ALDH1A1* gene did not alter the expression of Skp1 mRNA and protein, suggesting that Skp1 is positioned upstream to Aldh1 in the hierarchy of DAergic function. Consistent with this postulation is the observation that cells deficient in *SKP1A* levels or double knocked down for *ALDH1A1/SKP1A* are significantly more susceptible to serum deprivation than those deficient only in *ALDH1A1*. This difference likely reflects the strategic position of Skp1 in DAergic function, controlling survival pathways activated by neurotrophins and other protective factors present in serum, while the deficit of Aldh1 probably impairs a restricted molecular target/s downstream of Skp1.

Despite extensive research, the questions of how and why do SN neurons die remain unanswered. DA has long been implicated in the pathogenesis of PD. The reaction catalyzed by MAO to form DOPAL from DA produces hydrogen peroxide (H_2_O_2_), which can generate other ROS and free radicals. Furthermore, the content of MAO-B is increased in aged and PD brains, which further contributes to OS. Also, auto-oxidation of DA can lead to an array of toxic metabolites (Curtius et al. 1974; Spencer et al. 1998; Stokes et al. 1999) and generation of reactive oxygen species (ROS) (Li et al. 2001; Maguire-Zeiss et al. 2005). However, very high DA concentrations are required for toxicity in vitro (300 µM) and in vivo (77 µM) far exceeding physiologic concentrations of DA (Filloux and Townsend 1993). This suggests that it is more likely that a DA metabolite may be the culprit leading to toxicity. Consistent with this conjecture, Burke and colleagues (Burke et al. 1998; Li et al. 2001) have demonstrated that DOPAL generates the hydroxyl (OH) radical in the presence of H_2_O_2_. This effect was specific for DOPAL in that neither DA nor its metabolites homovanillic acid (HVA), DOPAC, 3,4-Dihydroxyphenylethanol (DOPET), or 3,4-dihydroxyphenylglycolaldehyde (DOPEGAL) generated OH radicals under the same conditions (Burke et al. 1998; Li et al. 2001). The same authors also reported that following stereotactic injections of DOPAL, dopamine, and other metabolites into the SN of Sprague–Dawley, only DOPAL, at concentrations within the physiological range of 2–3 μM, caused neurodegeneration (Burke et al. 2003).

DOPAL and H_2_O_2_ may interact with iron in the SN via the Fenton reaction to generate hydroxyl radicals, which can enter the mitochondria via the voltage-dependent anion channel and inhibit Complex I. Conversely, Complex I inhibitors (such as MPP^+^) also reduce the ability of Aldhs to remove DOPAL by reducing the availability of NAD^+^, a cofactor required for aldehyde dehydrogenase activity [(Galvin 2006); Fig. 18]. Consistent with this, our findings have shown that the deficiency of *ALDH1A1* in single or double knocked down clones along with preexisting mitochondrial dysfunction caused by MPP^+^ neurotoxin, acted synergistically to enhance cell death. This effect was seen even at a sub-effective concentration of the toxin, highlighting the important role played by Aldh1 in protecting SN DA-containing neurons from modest mitochondrial malfunction. In this context, Lamensdorf and colleagues (Lamensdorf et al. 2000) have reported that accumulation of DOPAL by both Complex I inhibition (rotenone) or *ALDH* inhibition potentiated rotenone-induced toxicity in PC-12 cells. Similarly, the silencing of Skp1 also exacerbated the damage caused by an injurious concentration of MPP^+^, probably reflecting an Aldh1-mediated effect. It cannot be ruled out; however, the participation of other pathways commanded by Skp1, in addition to that shared with Aldh1.

The above findings have important implication regarding the suggested role played by Skp1 in DAergic neuron function. They support the possibility that Skp1 orchestrates the concerted action of several survival biological processes in the DAergic neurons of SNpc; one of them being the detoxification of toxic aldehyde derivatives of DA, by a targeted modulation of Aldh1 expression/activity. PD is associated with elevated levels of DOPAL (Burke et al. 2003) and reduced detoxification of DOPAL from deficient Aldh1 function may be a contributing factor in the suggested neurotoxicity of this compound. Indeed, decreased expression of Aldh1 in the SNpc of PD individuals has been reported (Galter et al. 2003; Grunblatt et al. 2004). Unlike free radicals, aldehydes possess half-lives ranging from a few hours to days, allowing the aldehyde products to accumulate at the site of injury or disease, injuring neighboring healthy cells and slowly but progressively enlarging the lesion (Byrne 2003). More support in favor of the above assumption was provided by exposing either *SKP1A*- or *ALDH1A1*-silenced *SN4741* cells to exogenously administered DA. Under normal conditions, DA is metabolized by the consecutive action of MAO and Aldh to inert, non-harmful acidic products. However, the deficiency of either *SKP1A* or *ALDH1A1* increased the susceptibility to the damage caused by an excess of DA. The higher sensitivity was observed in the *ALDH1A1*-silenced *SN4741* cells, likely resulting from a direct interference with the mRNA machinery. Finally, the injury was significantly prevented by pretreatment of both cell clones with the MAO-B inhibitor, rasagiline, supplying further evidence to the notion that Skp1 plays a protective role in DAergic neuronal function, which involves a targeted action on DA-derived aldehydes clearance.

In conclusion, evidence has been provided for potential interacting effects of genetic reductions in Skp1 and Aldh1 (intrinsic/genetic predisposition), external stressors (serum withdrawal, excess of DA), and MPP^+^ neurotoxin.

### Deficiency of *SKP1A* in mouse SNpc replicates important features of PD

The main finding of the in vivo site-directed Skp1 silencing was a partial replication of histopathological and motor features of PD in the course of 3–8 months, after viral-mediated knock down of *SKP1A* in mouse. The reduction of the vertical movements, that is rearing, may be reminiscent of the early occurrence of hypokinesia and axial, postural instability in PD. This effect is associated with a decrease in the expression of Skp1 protein levels within DAergic neurons in the compacta. The deficiency caused a parallel drastic loss of TH-IR that was accompanied by a comparable decline of TH expression in the striatum. At this point, we do not have an index of the time-course of TH decline given that the results are from an end-stage assessment. Also, it is not clear whether the reduced TH-IR in the striatum results from loss of TH neurons in the SNpc, or reduced TH expression by the resident neurons or both. However, there are some indications that the damage to the neurons resulting from Skp1 deficiency may have been initiated at early stage: a progressive loss of terminal projections in the striatum, and second, the gradual age-dependent decline in the rearing behavior (vertical movement), suggests an early impairment in some motor aspects.

In summary, these studies were able to reproduce partial histopathological and motor features of PD in the course of 3–8 months after viral-mediated knockdown of *SKP1A*. It is possible that a more severe motor phenotype will develop beyond the 8 months period. Our findings support the view that this viral-mediated genetic model of PD may help to elucidate the role of Skp1 action in DAergic function and allow the screening of candidate therapeutic molecules.

The results in *SKP1A* deficient mice are clearly demonstrating time-dependent motor disabilities and robust loss of TH-IR in the SNpc and striatum. Future studies should contemplate intrinsic and environmental manipulations to better emulate the motor and non-motor disabilities, the progressive nature of PD, and striatal–nigral and adjacent areas pathology. One such intrinsic modifier is aging, considered the main risk factor for PD. Currently, lentiviral-mediated reduction of *SKP1A* is performed in the SNpc of aged mice. Genetic manipulation will consider knock down of genes assumed to cross-talk with *SKP1A* such as *ALDH1A1* and *PARK15/FBXO7* on top of *SKP1A*-deficient aged mice. This model of SPD will help to advance our understanding of the pathological course of the sporadic form of the disease in “real-time” mature mice and provide a valuable tool for the evaluation of drugs with potential “disease modifying activity”.

Conclusively, the present findings have identified Skp1 as a fundamental player in DA neuron function, differentiation, and survival. Future studies should contemplate intrinsic and environmental manipulations to better emulate the motor and non-motor disabilities, the progressive nature of PD, and striatal–nigral and adjacent areas pathology. If successful, the availability of a model of SPD that can replicate the adult-onset, progressive neurodegeneration in specific tissues, and motor deficits will provide a reliable “platform” to develop new therapeutic interventions.

## Abbreviations

AADC: Aromatic amino-acid decarboxylase
AD: Alzheimer disease
ADH5: Alcohol dehydrogenase 5
ALDH1A1: Aldehyde dehydrogenase 1 family, member A1
ARPP-21: AMP-regulated phosphoprotein
°C: Degree Celsius
cDNA: Complementary DNA
CDKs: Cyclin-dependent kinases
DA: Dopamine
DAergic: Dopaminergic
DMEM: Dulbecco’s modified Eagle’s medium
dsRNA: Double-stranded RNA
DNA: Deoxyribonucleic acid
DOPAC: 3, 4- Dihydroxyphenylacetic acid
EDTA: Ethylenediaminetetraacetic acid
EGLN1: Egl nine homolog 1 (C. elegans)
FACS: Flow cytometry analysis
FCS: Fetal calf serum
h: Hour
HIF: Hypoxia-inducible factor
HIV-1: Human immunodeficiency virus type 1
H_2_O_2_: Hydrogen peroxide
IHC: Immunohistochemistry
IRP2: Iron regulatory protein 2
LBs: Lewy bodies
L-DOPA: Levodopa
LRKK2: Leucine-rich repeat kinase 2
LVs: Lentiviruses
MAO: Monoamine oxidase
μg: Microgram
μM: Micromolar
ml: Milliliter
mM: Millimolar
MPTP: N-methyl-4-phenyl-1,2,3,6-tetrahydropyridine
MPP +: 1-Methyl-4-phenylpyridinium ion
mRNA: Messenger RNA
MTT 3: (4,5-Dimethylthiazol-2-yl)-2,5-diphenyl tetrazolium bromide
OS: Oxidative stress
PAGE: Polyacrylamide gel electrophoresis
PBS: Phosphate-buffered saline
PCR: Polymerase chain reaction
PD: Parkinson’s disease
PET: Positron emission tomography
PHD2: Proline hydroxylase-2
pI: Propidium iodide
PINK-1: PTEN- induced kinase 1
PSMC4: Proteasome 26S subunit, ATPase, 4
Rasagiline: N-propargyl-1R-aminoindan
Rb: Retinoblastoma
RISC: Rnai silencing complex
RNA: Ribonucleic acid
ROS: Reactive oxygen species
RT: Reverse transcription
RT: Room temperature
pol-III: Polymerase III
SCFSkp1: Cullin-F-box protein
SDS: Sodium dodecyl sulfate
siRNA: Small interfering rnas
shRNA: Small-hairpin interfering rnas
SKP1: S-phase kinase-associated protein 1
SN: Substantia nigra
SNpc: Substantia nigra pars compacta
SNARE: Soluble NSF attachment protein receptor
SPECT: Single photon emission computed tomography
STX1A: Syntaxin-1A
SYNGR3: Synaptogyrin 3
SYT1: Synaptotagmin
TfR: Transferrin receptor
TH: Tyrosine hydroxylase
TBST: Tbs + tween 20
UCHL-1: Ubiquitin C-terminal hydrolase-L1
UPSU: Ubiquitin proteasome system
VMAT2: Vesicular monoamine transporter-2
VTA: Ventral tegmental area
vs: Versus
6-OHDA: 6-Hydroxydopamine
